# Associations of Greenness, Parks, and Blue Space With Neurodegenerative Disease Hospitalizations Among Older US Adults

**DOI:** 10.1001/jamanetworkopen.2022.47664

**Published:** 2022-12-20

**Authors:** Jochem O. Klompmaker, Francine Laden, Matthew H. E. M. Browning, Francesca Dominici, Marcia P. Jimenez, S. Scott Ogletree, Alessandro Rigolon, Antonella Zanobetti, Jaime E. Hart, Peter James

**Affiliations:** 1 Department of Environmental Health, Harvard T. H. Chan School of Public Health, Boston, Massachusetts; 2 Channing Division of Network Medicine, Department of Medicine, Brigham and Women’s Hospital and Harvard Medical School, Boston, Massachusetts; 3 Department of Epidemiology, Harvard T. H. Chan School of Public Health, Boston, Massachusetts; 4 Department of Parks, Recreation and Tourism Management, Clemson University, Clemson, South Carolina; 5 Department of Biostatistics, Harvard T. H. Chan School of Public Health, Boston, Massachusetts; 6 Department of Epidemiology, Boston University School of Public Health, Boston, Massachusetts; 7 OPENspace Research Centre, School of Architecture and Landscape Architecture, University of Edinburgh, Edinburgh, United Kingdom; 8 Department of City and Metropolitan Planning, University of Utah, Salt Lake City; 9 Department of Population Medicine, Harvard Medical School and Harvard Pilgrim Health Care Institute, Boston, Massachusetts

## Abstract

**Importance:**

Exposure to natural environments has been associated with health outcomes related to neurological diseases. However, the few studies that have examined associations of natural environments with neurological diseases report mixed findings.

**Objective:**

To evaluate associations of natural environments with hospital admissions for Alzheimer disease and related dementias (ADRD) and Parkinson disease (PD) among older adults in the US.

**Design, Setting, and Participants:**

This open cohort study included fee-for-service Medicare beneficiaries aged 65 years or older who lived in the contiguous US from January 1, 2000, to December 31, 2016. Beneficiaries entered the cohort on January 1, 2000, or January 1 of the year after enrollment. Data from US Medicare enrollment and Medicare Provider Analysis and Review files, which contain information about individual-level covariates and all hospital admissions for Medicare fee-for-service beneficiaries, were analyzed between January 2021 and September 2022.

**Exposures:**

Differences in IQRs for zip code–level greenness (normalized difference vegetation index [NDVI]), percentage park cover, and percentage blue space cover (surface water; ≥1.0% vs <1.0%).

**Main Outcomes and Measures:**

The main outcome was first hospitalizations with a primary or secondary discharge diagnosis of ADRD or PD. To examine associations of exposures to natural environments with ADRD and PD hospitalization, we used Cox-equivalent Poisson models.

**Results:**

We included 61 662 472 and 61 673 367 Medicare beneficiaries in the ADRD and PD cohorts, respectively. For both cohorts, 55.2% of beneficiaries were women. Most beneficiaries in both cohorts were White (84.4%), were not eligible for Medicaid (87.6%), and were aged 65 to 74 years (76.6%) at study entry. We observed 7 737 609 and 1 168 940 first ADRD and PD hospitalizations, respectively. After adjustment for potential individual- and area-level confounders (eg, Medicaid eligibility and zip code–level median household income), NDVI was negatively associated with ADRD hospitalization (hazard ratio [HR], 0.95 [95% CI, 0.94-0.96], per IQR increase). We found no evidence of an association of percentage park and blue space cover with ADRD hospitalization. In contrast, NDVI (HR, 0.94 [95% CI, 0.93-0.95], per IQR increase), percentage park cover (HR, 0.97 [95% CI, 0.97-0.98], per IQR increase), and blue space cover (HR, 0.97 [95% CI, 0.96-0.98], ≥1.0% vs <1.0%) were associated with a decrease in PD hospitalizations. Patterns of effect modification by demographics differed between exposures.

**Conclusions and Relevance:**

The findings of this cohort study suggest that some natural environments are associated with a decreased risk of ADRD and PD hospitalization.

## Key Points

**Question:**

Are measures of the natural environment associated with hospitalization for Alzheimer disease and related dementias (ADRD) and Parkinson disease (PD) among older individuals?

**Findings:**

In this US-based cohort study of approximately 62 million Medicare beneficiaries aged 65 years or older, protective associations of greenness (normalized difference vegetation index), park cover, and blue space cover with PD hospitalization were observed. Greenness, but not park or blue space cover, was associated with a decreased risk of ADRD hospitalization.

**Meaning:**

These findings suggest that exposure to some natural environments may reduce the risk of ADRD and PD hospitalization among older adults.

## Teaser

This cohort study explores the association of natural environments—using measures of greenness, parks, and blue space—with hospitalizations for Alzheimer disease and related dementias and Parkinson disease among older individuals in the US.

## Introduction

Neurological disorders are the leading cause of disability and the second leading cause of death worldwide.^1^ The most prevalent neurological diseases in the US are Alzheimer disease and related dementias (ADRD) and Parkinson disease (PD).^2,3^ The estimated direct cost of ADRD and PD in the US is around $321 and $25 billion per year, respectively.^2,4^ The prevalence of neurological diseases will likely continue to increase due to lengthening life expectancy.^1^ No cures exist for ADRD or PD, so it is important to identify modifiable risk factors.

Environmental exposures may affect the risk and/or exacerbate symptoms of ADRD and PD.^5,6^ Air pollution has been linked to ADRD and PD incidence^7,8^; less is known about effects of natural environments, which may impact ADRD and PD incidence or symptoms through several mechanisms. Natural environments, such as forests, parks, street trees, and rivers, can help reduce stress and restore attention, provide settings for physical activity and social interactions, and may reduce exposure to air pollution, extreme heat, and noise.^9,10^ These exposures may also protect against several neurological-related outcomes such as cognitive decline, stroke, and neurodegenerative disease mortality.^11,12,13,14^ Studies have examined associations of green space with ADRD and PD incidence but reported mixed findings.^15,16,17^

We aimed to evaluate the associations of 3 natural environment measures (greenness, park cover, and blue space cover) with first ADRD and PD hospital admissions in a cohort of Medicare beneficiaries (approximately 61.7 million individuals). ADRD and PD hospital admissions differ from disease onset, and potential associations with first ADRD and PD hospitalizations should be interpreted as a measure of accelerated or slower disease progression or increased or decreased susceptibility.^8,18,19^ As some studies suggest that associations of natural environments with health outcomes are greater in urban areas, we specifically evaluated associations in urban zip codes (≥1000 persons/square mile). Additionally, we assessed if associations were modified by demographics.

## Methods

The protocol for this cohort study was approved by the Human Subjects Committee of the Harvard T. H. Chan School of Public Health. This study was deemed exempt from informed consent requirements because previously collected administrative data were used. The study followed the Strengthening the Reporting of Observational Studies in Epidemiology (STROBE) reporting guideline.

### Study Population

Medicare is the US federal government’s health insurance program for younger individuals with disability and for individuals aged 65 years or older. We included all fee-for-service Medicare beneficiaries who lived in the contiguous US from January 1, 2000, through December 31, 2016, and were aged 65 years or older. Data were obtained from the Medicare enrollment and Medicare Provider Analysis and Review files. Beneficiaries entered the cohort on January 1, 2000, or on January 1 of the year after enrollment. They were followed until first hospital admission for the outcome of interest or until they were censored, reached the end of follow-up (December 31, 2016), or died, whichever occurred first. Separate cohorts for ADRD and PD hospitalizations were created.

### Outcome Definition

Hospital admissions were defined by *International Classification of Diseases, Ninth Edition* (*ICD-9*) codes from 2000 through the third quarter of 2015. From the fourth quarter of 2015 onward, *International Statistical Classification of Diseases, Tenth Revision* (*ICD-10*) codes were used. We used *ICD* codes specified by the Chronic Conditions Data Warehouse to define ADRD hospitalization.^20^ We looked at first hospital admissions with a primary or secondary discharge diagnosis of ADRD or PD. The eMethods in Supplement 1 lists the *ICD-9* and *ICD-10* codes used to define ADRD and PD.

### Exposure Assessment

As we only had information about the residential zip code of each beneficiary, we calculated zip code–level exposures. Detailed information about the exposures can be found in the eMethods in Supplement 1. We calculated the normalized difference vegetation index (NDVI) by using images from Landsat 7-8 (US Geological Survey) with 30-m^2^ resolution. The NDVI is an indicator of greenness calculated as the ratio between red and near-infrared values.^21^ The NDVI ranges from −1 to 1, with larger values indicating higher levels of live vegetation and negative values corresponding to water. After setting negative values to 0, the spatially weighted mean summer NDVI (June 1 to August 31) was calculated for each zip code in the US for each year from 2000 to 2016 (eFigure 1 in Supplement 1).

Park exposure was based on the US Geological Survey Protected Areas Database of the US version 2.1. All land types likely to be known and used by the general public for outdoor recreation were selected to create a park cover data set. We calculated the percentage park cover for each zip code.

Blue space was based on the Joint Research Centre Global Surface Water data set.^22^ This data set contains information about the location and temporal distribution of surface water, based on Landsat images from 1984 to 2018. As zip codes are used for postal delivery services, adjacent water bodies are not always included in zip code boundaries. Therefore, we calculated spatially weighted mean blue space values for zip codes and 1000-m buffers around their perimeter. Given the limited variability of the distribution of percentage blue space cover, we used a binary blue space cover indicator (dichotomized at 1.0%). To determine the sensitivity of our results to this cut point, we also evaluated 5.0% as a cut point.

### Covariates

Detailed information on covariates is located in the eMethods in Supplement 1. Briefly, we obtained data on age, sex, race and ethnicity (Black, White, other [including American Indian or Alaska Native, Asian, Hispanic, and other race or ethnicity], or unknown race or ethnicity), Medicaid eligibility (a proxy for low socioeconomic status [SES]), year of entry, and zip code of residence for all Medicare beneficiaries. Race and ethnicity information of Medicare beneficiaries is generally obtained from the US Social Security Administration, which collects data at the time of application for a Social Security Number.^23^ We included several zip code–level covariates from the US Census and the American Community Survey and county-level smoking status from the nationwide Behavioral Risk Factor Surveillance System. Further, we used the 4 US Census regions and 9 divisions (eFigure 2 in Supplement 1) to adjust for regional differences not accounted for by other variables. Previous studies reported regional differences in ADRD and PD prevalence that may be related to differences in the recognition of symptoms and willingness to document the disease.^24,25^ We also linked other environmental exposures including zip code–level particulate matter less than 2.5 μm (PM_2.5_), nitrogen dioxide (NO_2_), and meteorological factors (maximum daily temperature, specific humidity, and precipitation).

### Statistical Analysis

Data were analyzed between January 2021 and September 2022. To examine associations of each exposure with first ADRD and PD hospitalizations, we used a Cox-equivalent reparameterized Poisson approach (described in detail elsewhere^19^). First, we aggregated beneficiaries who lived within the same zip code in each year by the following: sex, 2-year age categories at study entry, race and ethnicity, Medicaid eligibility, and year of follow-up. Next, we fitted quasi-Poisson models with count of first ADRD or PD hospitalizations as the dependent variable and total person-time of beneficiaries as the offset. This model is mathematically equivalent to a time-varying Cox proportional hazards model under an Anderson-Gill representation but is more computationally efficient. We applied an m-out-n bootstrap method using zip code units to calculate statistically robust CIs.

We examined associations in the full population and in the subset of urban zip codes (zip code population density ≥1000 persons/square mile). We fitted models with increasing degrees of covariate adjustment to evaluate the impact of potential confounders. In our main model, we included all 3 types of natural environment exposures simultaneously and adjusted for the following: calendar year, region, US Census zip code–level covariates, county-level smoking status, an offset for total person-time, and strata for all possible combinations of age, sex, race and ethnicity, Medicaid eligibility, and follow-up year to allow for flexible strata-specific baseline rates. We included race and ethnicity in our models as a proxy for life experiences of racism and marginalization.^26^ The shape of the exposure-response curves for greenness and park cover was examined using natural splines with 2 to 3 degrees of freedom. We performed stratified analyses by sex, age, race and ethnicity, Medicaid eligibility, region, and neighborhood SES measures (median household income, median home value, and percentage of the population below the poverty level). Hazard ratios (HRs) for NDVI and percentage park cover are expressed per IQR difference. Hazard ratios of blue space cover (≥1.0%) are given compared with the reference category (<1.0% blue space cover).

For sensitivity analyses, we ran single-exposure models, modeled the alternative blue space cut point (5.0%), and additionally adjusted for PM_2.5_, NO_2_, and meteorological factors. Further, we ran models with adjustment for US Census division instead of region. We excluded individuals who had their first hospital admission within the first year of their follow-up and all records in 2000 to eliminate potential prevalent cases. To assess the impact of increasing natural environment exposures, we estimated the total number of hospital admissions avoided among this population in 1 year if, hypothetically, zip code NDVI and park cover increased by 0.5 IQR and blue space cover would be 1.0% or greater (eMethods in Supplement 1).

Beneficiaries with missing data in any variables included in the main model were removed from the cohort (approximately 2.1%). R software version 3.6.1 (R Project for Statistical Computing) was used for analyses, and analyses were conducted on the Harvard Research Computing Environment supported by the Institute for Quantitative Social Science at Harvard University. Two-sided *P* < .05 was considered statistically significant.

## Results

The ADRD and PD cohorts included 61 662 472 and 61 673 367 Medicare beneficiaries, respectively. For both cohorts, 55.2% of beneficiaries were women and 44.8% were men; 8.8% were Black, 84.4% were White, and 6.8% were of another racial or ethnic minority group or were of unknown race or ethnicity. In addition, most beneficiaries in both cohorts were not eligible for Medicaid (87.6%) and were aged 65 to 74 years (76.6%) at study entry (Table 1). In the full cohort, we observed 7 737 609 first ADRD hospital admissions in 451 389 159 person-years and 1 168 940 first PD hospital admissions in 465 039 445 person-years. In the urban ADRD and PD cohorts, we observed 3 876 310 first ADRD hospital admissions in 211 928 582 person-years and 574 742 first PD hospital admissions in 218 823 939 person-years. Maps of ADRD and PD hospitalizations per 100 000 person-years are shown in eFigure 3 in Supplement 1. In the full cohort, the median (IQR) NDVI was 0.52 (0.27), the median percentage park cover was 7.4 (15.8), and 40.8% had blue space cover (≥1.0%). In the urban cohort, the median NDVI was lower, while the median park cover was higher than in the full cohort (eTables 1 and 2 in Supplement 1). Percentage park cover was negatively correlated with NDVI in the full cohort (Spearman ρ = −0.23) (eFigure 4 in Supplement 1) and was not correlated with NDVI in the urban cohort (Spearman ρ = 0.03).

**Table 1.** Descriptive Statistics of All US Medicare Fee-for-Service Beneficiaries Included in the Alzheimer Disease and Related Dementias Cohort From 2000 Through 2016

Demographic at study entry	Values
Total No. of beneficiaries	61 662 472
**Individual-level covariate, No. (%)**	
Sex	
Female	34 010 915 (55.2)
Male	27 651 557 (44.8)
Age, y	
65-74	47 237 464 (76.6)
75-84	10 583 080 (17.2)
≥85	3 841 928 (6.2)
Race and ethnicity	
Black	5 421 282 (8.8)
White	52 044 489 (84.4)
Other^a^	4 196 701 (6.8)
Medicaid eligibility	
Not eligible	53 991 858 (87.6)
Eligible	7 670 614 (12.4)
US region	
Northeast	12 354 729 (20.0)
South	23 483 516 (38.1)
Midwest	14 656 222 (23.8)
West	11 168 005 (18.1)
**Zip code–level covariate, median (IQR)^b^**	
Natural environment measures	
NDVI	0.52 (0.36-0.63)
Park cover, %	7.8 (2.2-18.0)
Blue space cover (1000-m buffer), %	0.5 (0.1-3.4)
Aggregated data with ≥1.0% blue space cover, %	41.4
US Census covariates and American Community Survey	
Population density (persons/square mile)	608.2 (84.3-3096.3)
Median home value ($1000)	144.0 (92.2-239.5)
Median household income ($1000)	46.7 (36.6-61.9)
With less than a high school degree, %	24.3 (15.1-36.4)
Below the poverty level, %	8.5 (5.4-13.4)
Owner-occupied housing units, %	71.5 (58.8-80.6)
Black race, %	3.9 (0.8-14.5)
Hispanic ethnicity, %	5.3 (1.8-16)
BRFSS covariate	
Ever smoked, %	46.2 (41.5-50.5)
Other environmental exposures	
Summer temperature, °C	29.8 (27.3-32.5)
Summer specific humidity, g of water vapor/kg of dry air	12.0 (10.4-14.4)
Summer total precipitation (daily total), mm	3.1 (1.9-4.2)
PM_2.5_, μg/m^3^	9.7 (7.8-11.8)
NO_2_, ppb	16.6 (10.9-24.8)

Abbreviations: BRFSS, Behavioral Risk Factor Surveillance System; NDVI, normalized difference vegetation index; NO_2_, nitrogen dioxide; PM_2.5_, particulate matter <2.5 µm.

^a^ Includes American Indian or Alaska Native, Asian, Hispanic, and other or unknown race or ethnicity.

^b^ Descriptive statistics of the zip code–level covariates are given for the strata (aggregated data for 2000-2016 based on zip code, year, sex, race and ethnicity, Medicaid eligibility, 2-year categories of age at study entry and year of follow-up). Descriptive statistics of the zip code–level covariates for the Parkinson Disease cohort are shown in eTable 1 in Supplement 1.

### Associations in the Full Cohort

Exposure-response curves showed generally linear associations for percentage park cover and NDVI (eFigure 5 in Supplement 1). The NDVI was associated with a decrease in ADRD hospitalizations after adjustment for potential confounders (HR, 0.95 [95% CI, 0.94-0.96], per IQR increase) (Table 2). We found no evidence of an association of percentage park cover and blue space cover (≥1.0%) with ADRD hospitalization. All 3 exposures were associated with a decrease in PD hospitalizations after adjustment for potential confounders. Associations of the exposures with ADRD and PD hospitalizations in minimally adjusted models were generally similar to associations in fully adjusted models (eFigure 6 in Supplement 1).

**Table 2.** Hazard Ratios of NDVI, Percentage Park Cover, and Blue Space Cover of 1.0% or Greater With Hospitalization for ADRD and PD^a^

Exposure	ADRD hospitalization			PD hospitalization	
	Full cohort	Urban cohort	Full cohort		Urban cohort
NDVI	0.95 (0.94-0.96)	0.99 (0.98-1.01)	0.94 (0.93-0.95)		0.95 (0.93-0.97)
Park cover, %	0.99 (0.99-1.00)	0.99 (0.98-1.00)	0.97 (0.97-0.98)		0.99 (0.98-1.00)
Blue space cover ≥1.0%, %	0.99 (0.99-1.00)	1.00 (0.99-1.01)	0.97 (0.96-0.98)		0.98 (0.97-1.00)

Abbreviations: ADRD, Alzheimer disease and related dementias; NDVI, normalized difference vegetation index; PD, Parkinson disease.

^a^ Data are presented as hazard ratio (95% CI). Associations of NDVI (0.27) and percentage park cover (15.9) are expressed per IQR increase of the full cohort. Associations of blue space cover (≥1.0%) are given compared with the reference category (<1.0% blue space cover). Models are described in the Statistical Analysis section.

**Figure 1.**
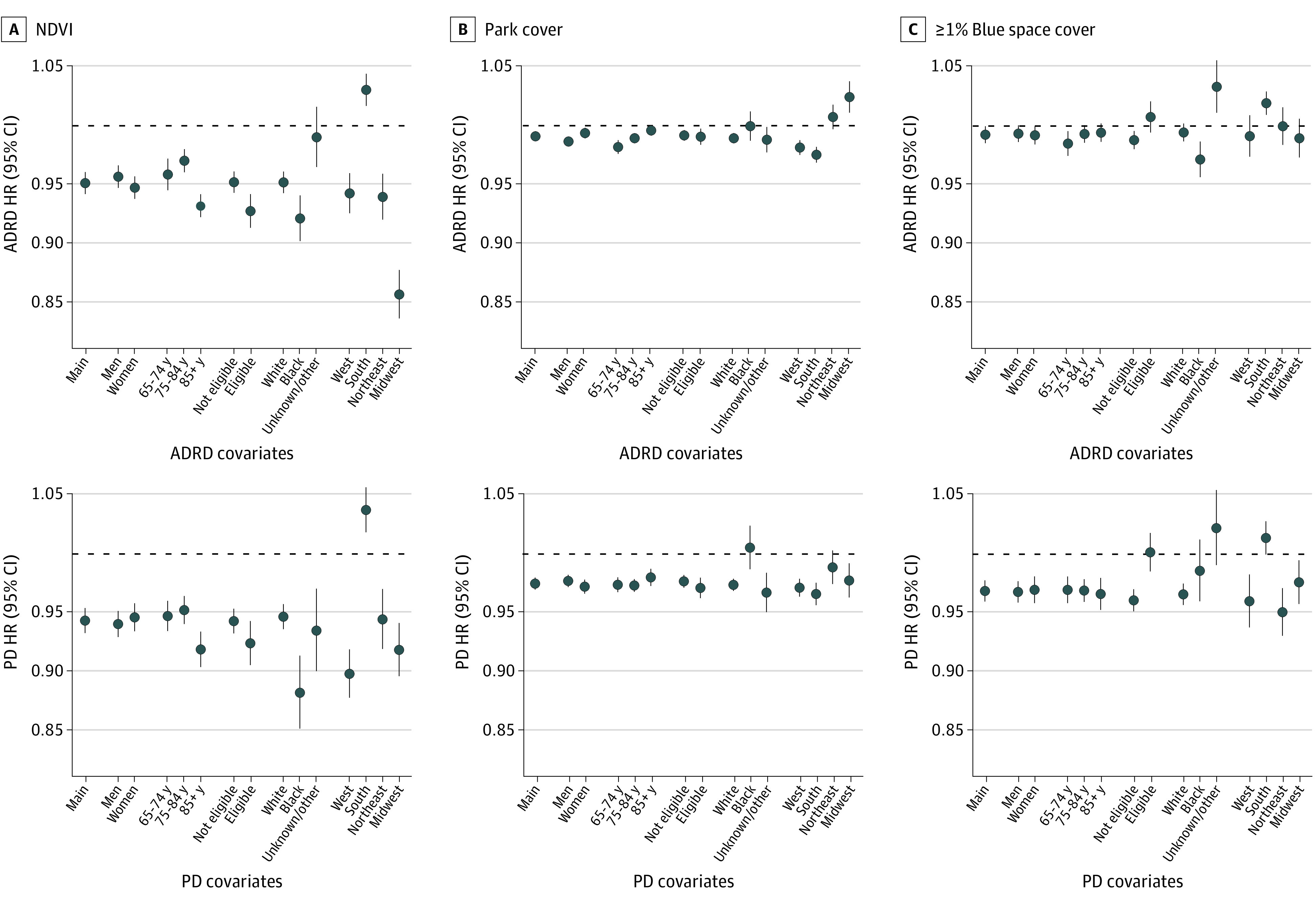
Associations of Normalized Difference Vegetation Index (NDVI), Percentage Park Cover, and Blue Space Cover (≥1.0%) With Hospitalization for Alzheimer Disease and Related Dementias (ADRD) and Parkinson Disease (PD) in the Full Medicare Cohort in Stratified Analyses A, Associations of normalized difference vegetation index (NDVI) are expressed per IQR increase of the full cohort (0.27). B, Associations of percentage park cover are expressed per IQR increase of the full cohort (15.9). C, Associations of blue space cover (≥1.0%) are given compared to the reference category (<1.0% blue space cover). Models included NDVI, percentage park cover, and percentage blue space cover and were adjusted for calendar year, region, US Census covariates, percentage ever smoked, an offset for total person-time, and strata for all possible combinations of sex, race and ethnicity, Medicaid eligibility, age at study entry (2-year categories), and follow-up year. HR indicates hazard ratio.

**Figure 2.**
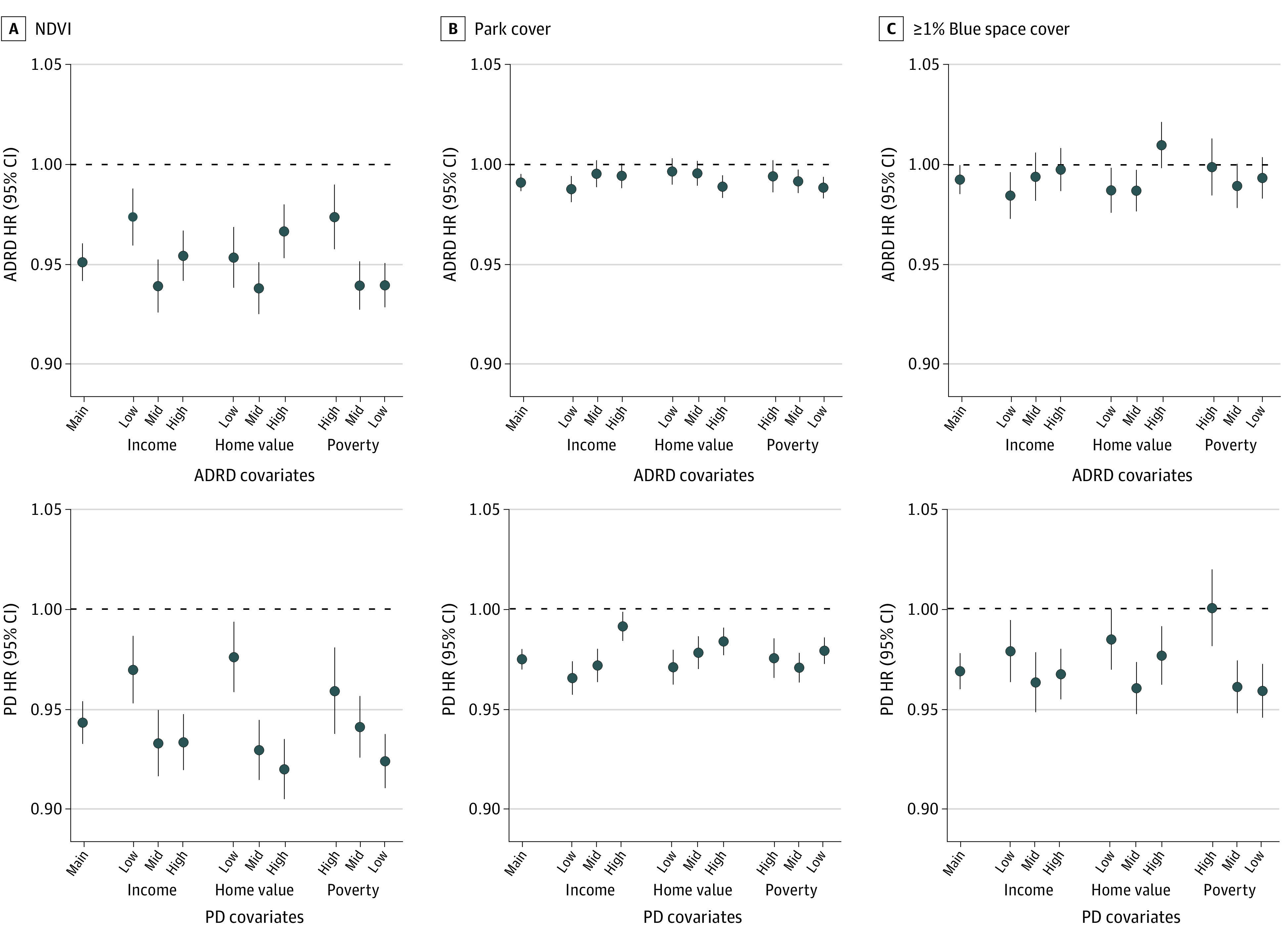
Associations of Normalized Difference Vegetation Index (NDVI), Percentage Park Cover, and Blue Space Cover (≥1.0%) With Hospitalization for Alzheimer Disease and Related Dementias (ADRD) and Parkinson Disease (PD) in the Full Medicare Cohort in Stratified Analyses by Neighborhood Socioeconomic Status A, Associations of normalized difference vegetation index (NDVI) are expressed per IQR increase of the full cohort (0.27). B, Associations of percentage park cover are expressed per IQR increase of the full cohort (15.9). C, Associations of blue space cover (≥1.0%) are given compared to the reference category (<1.0% blue space cover). Models included NDVI, percentage park cover, and percentage blue space cover and were adjusted for calendar year, region, US Census covariates, percentage ever smoked, an offset for total person-time, and strata for all possible combinations of sex, race and ethnicity, Medicaid eligibility, age at study entry (2-year categories), and follow-up year. Income indicates median household income, home value denotes median home value, and poverty is the percentage below the poverty level. To define strata, we used the following quantiles (q33.3, q66.7) for the ADRD cohort: median household income ($1000): 40.0, 55.6; median home value ($1000): 107.3, 195.6; and percentage below the poverty level: 6.4, 11.4. For the PD cohort, we used the following quantiles: household income ($1000): 40.0, 55.7; median home value ($1000): 107.6, 196.0; and percentage below the poverty level: 6.4, 11.4.

Patterns of effect modification by individual demographics and region differed between exposures (Figure 1). For Black individuals, we observed no evidence of associations of percentage park cover with ADRD and PD hospitalization, whereas we observed protective associations of NDVI with ADRD (HR, 0.92 [95% CI, 0.90-0.94] per IQR increase) and PD (HR, 0.88 [95% CI, 0.85-0.91] per IQR increase) hospitalizations. In the South, NDVI and blue space cover (≥1.0%) were positively associated, while percentage park cover was negatively associated with both outcomes. Further, the protective associations of NDVI with both outcomes were greatest among Medicaid-eligible individuals, whereas protective associations of blue space cover (≥1.0%) were only observed for noneligible individuals (PD HR, 0.96 [95% CI, 0.95-0.97], ≥1.0% vs <1.0%). Patterns of effect modification by neighborhood SES also differed between exposures (Figure 2). For PD hospitalization, associations of percentage park cover were greatest in low-SES zip codes, while associations of NDVI were greatest in mid- and high-SES zip codes.

### Associations in the Urban Cohort

We found no evidence for associations of NDVI, percentage park cover, and blue space cover (≥1.0%) with ADRD hospitalization in the urban cohort (Table 2). Both NDVI and blue space cover (≥1.0%) were associated with a decrease in PD hospitalizations. Similar to what we observed in the full cohort, patterns of effect modification by individual demographics, region (eFigure 7 in Supplement 1), and neighborhood SES (eFigure 8 in Supplement 1) differed between exposures. We observed protective associations of percentage park cover with PD hospitalization for Medicaid-eligible individuals, individuals of unknown or other race or ethnicity, and individuals living in low-SES neighborhoods. Associations of blue space cover (≥1.0%), on the other hand, were positive for Medicaid-eligible individuals, individuals of unknown or other race or ethnicity, and individuals living in high-poverty neighborhoods. We also observed protective associations of percentage park cover in the South but not in other regions. Associations of NDVI were null (PD hospitalization) or weakly positive (ADRD hospitalization) in the South and protective elsewhere.

### Sensitivity Analysis

Associations of our sensitivity analyses are shown in eTables 3 and 4 in Supplement 1. Associations of all 3 environmental exposures generally attenuated after adjustment for air pollutants or meteorological indicators. Assuming that modeling assumptions are correct, that the associations we have observed are true, and that hypothetically, zip code NDVI and park cover increased by 0.5 IQR and blue space cover increased to 1.0% or greater in all zip codes, we would expect 15 424 fewer ADRD hospital admissions and 3982 fewer PD hospital admissions each year (eTable 5 in Supplement 1).

## Discussion

The results of this cohort study suggest protective associations of greenness, percentage park cover, and blue space cover (≥1.0%) with PD hospitalization and of greenness with ADRD hospitalization in the full and urban cohorts. In line with our study, Yu et al^16^ recently observed protective associations of greenness with PD incidence in a cohort of Chinese urban individuals aged older than 18 years. Yuchi et al^15^ found protective associations of greenness with PD incidence in a cohort of 45- to 85-year-old urban Canadians. Previous studies that evaluated associations of green space with ADRD incidence have reported mixed findings. Yuchi et al^15^ found indications of a protective effect of greenness on non-Alzheimer dementia incidence, but reported positive associations with Alzheimer disease incidence. Slawsky et al^17^ found protective associations of green space with incident vascular dementia and mixed pathologies in a cohort of Americans aged 75 years; associations with incident all-cause dementia and Alzheimer disease showed nonlinear trends. Differences in associations could be due to variations in study population, covariate adjustment, exposure assessment, and outcome definition.

We observed that associations of greenness with ADRD and PD attenuated but remained after adjustment for air pollutants. Associations of percentage park cover with PD hospitalization were close to 0 after adjustment for air pollutants. Air pollutants could be potential confounders, but attenuations could also indicate potential mediation effects. Other potential pathways could be stress reduction, attention restoration, increased physical activity, and social interactions. Physical activity and social interactions have been shown to be beneficial for cognitive function, dementia, and PD.^27,28,29,30^ Studies also linked natural environments to a reduced risk of depression,^31,32,33^ which has also been associated with an increased risk of ADRD and PD.^34,35^ Moreover, studies have linked natural environments to a reduced risk of cardiometabolic diseases,^36,37,38^ which are important risk factors for ADRD.^39^ The protective association of NDVI, but not of park and blue space cover, with ADRD hospitalization could be due to greater associations of NDVI with pathways that affect ADRD, such as air pollution.

We did not observe greater associations in the urban cohort than the full cohort. This contradicts a review showing that associations of green space with health were typically greater in urban areas, though this review did not include neurodegenerative health outcomes.^40^ Differences in associations between the urban and full populations and between other strata could indicate differences in susceptibility to these exposures. However, these differences could also be due to variations in exposure levels, measurement error, or differences in ADRD and PD patterns of diagnosis. Studies reported differences in symptom recognition and willingness to document and diagnose the disease based on patient cultural and/or physician practice norms.^24,25^ As no cures exist for either disease, some individuals (and physicians) may not see the added value of obtaining a diagnosis. Different state policies, such as elder services funding and community outreach, could also affect ADRD and PD diagnosis rates.^24^

Patterns of effect modification by SES and race differed between exposures. In urban areas, associations of park cover, but not greenness or blue space, were generally greater for individuals from lower-SES neighborhoods, including Medicaid-eligible individuals and individuals living in low-SES neighborhoods. This could be because greenness and blue space in higher-SES neighborhoods may represent higher qualities of natural environments than in low-SES neighborhoods (eg, greenness on vacant lots, blue space close to harbors/air pollution sources) or because individuals from low-SES neighborhoods tend to use parks more often than other individuals.^41^ Also, we used a relatively coarse race indicator, which may have affected the stratified analyses.

A major strength of this study is the large study population and assessment of greenness, park cover, and blue space for each zip code in the contiguous US. All 3 exposures were included simultaneously to estimate associations independent of the other exposures. Medicare data include a fairly representative cohort of older adults in the US.

### Limitations

A limitation of this study is that we only had information on hospital records for fee-for-service beneficiaries; we may have missed some hospitalizations for beneficiaries who switched to managed care plans and back. We note that ADRD and PD have an insidious disease onset and neither requires hospital admission for diagnosis and treatment. Hospital admissions likely occur at more advanced disease stages. Hence, we did not evaluate associations with true onset of ADRD or PD but rather first hospital admission, which can be interpreted as accelerated disease progression (exacerbation of symptoms). However, our findings are consistent with existing studies of natural environments with ADRD and PD incidence. As we lacked information about the exact residential address of each beneficiary, we assessed zip code–level exposures, which likely resulted in measurement error. We did not have park cover and blue space cover data temporally matched for every year of follow-up. Hence, we assumed that the spatial distribution did not differ over time. As most parks (protected areas) and blue spaces do not change rapidly over time,^42^ we believe this is a reasonable assumption. No information about the quality and safety of parks, greenness, and blue spaces was available. We adjusted for only 1 individual-level SES measure (Medicaid eligibility) and no lifestyle factors, which may have resulted in an overestimation of the observed associations. However, models were adjusted for county-level smoking status and 8 zip code–level SES measures that are likely related to individual SES.

## Conclusions

The findings of this cohort study suggest that exposure to some natural environments may be protective for ADRD and PD hospitalization. As life expectancy increases globally, policy makers should consider interventions of natural environments to prevent ADRD and PD.

## Notes

Supplement 1.
eMethods.

eReferences
**eTable 1.** Descriptive Statistics of the Alzheimer Disease and Related Dementias and Parkinson Disease Hospitalization Cohorts, 2000 to 2016 **eTable 2.** Descriptive Statistics of Normalized Difference Vegetation Index, Percentage Park Cover, and Percentage Blue Space Cover in the Alzheimer Disease and Related Dementias and Parkinson Disease Hospitalization Cohorts (Aggregated Data), 2000 to 2016 **eTable 3.** Sensitivity Analyses of Normalized Difference Vegetation Index, Percentage Park Cover, and Blue Space Cover (≥1.0%) With Alzheimer Disease and Related Dementias and Parkinson Disease Hospitalization **eTable 4.** Sensitivity Analyses of Percentage Blue Space Cover Cutoffs With Alzheimer Disease and Related Dementias and Parkinson Disease Hospitalization **eTable 5.** Total Number of Alzheimer Disease and Related Dementias and Parkinson Disease Hospital Admissions Avoided in 1 Year **eFigure 1.** Spatial Variation of Zip Code−Level Normalized Difference Vegetation Index (2008), Percentage Park Cover, and Percentage Blue Space Cover in the Contiguous US **eFigure 2.** US Census Regions and US Census Divisions **eFigure 3.** Maps of Alzheimer Disease and Related Dementias and Parkinson Disease Hospitalizations per 100 000 Person-Years, 2000 to 2016 **eFigure 4.** Spearman Correlations Among Normalized Difference Vegetation Index, Percentage Park Cover, Percentage Blue Space Cover, and Other Covariates **eFigure 5.** Exposure-Response Curves (1, 2, or 3 Degrees of Freedom) of Normalized Difference Vegetation Index and Percentage Park Cover With Alzheimer Disease and Related Dementias and Parkinson Disease Hospitalization **eFigure 6.** Associations of Normalized Difference Vegetation Index, Percentage Park Cover, and Blue Space Cover (≥1.0%) With Alzheimer Disease and Related Dementias and Parkinson Disease Hospitalization in Models With Increasing Adjustment for Potential Confounders **eFigure 7.** Associations of Normalized Difference Vegetation Index, Percentage Park Cover, and Blue Space Cover (≥1.0%) With Alzheimer Disease and Related Dementias and Parkinson Disease Hospitalization in the Urban Population in Stratified Analyses **eFigure 8.** Associations of Normalized Difference Vegetation Index, Percentage Park Cover, and Blue Space Cover (≥1.0%) With Alzheimer Disease and Related Dementias and Parkinson Disease Hospitalization in the Urban Population in Stratified Analyses by Neighborhood Socioeconomic Status

Supplement 2.
Data Sharing Statement

## References

[1] Feigin VL, Nichols E, Alam T, ; GBD 2016 Neurology Collaborators. Global, regional, and national burden of neurological disorders, 1990-2016: a systematic analysis for the Global Burden of Disease Study 2016. Lancet Neurol. 2019;18(5):459-480. doi:10.1016/S1474-4422(18)30499-X 30879893 PMC6459001

[2] Alzheimer’s Association. Alzheimer’s Facts and Figures Report. 2021. Accessed January 4, 2022. https://www.alz.org/alzheimers-dementia/facts-figures

[3] Parkinson’s Foundation. Statistics. 2022. Accessed January 4, 2022. https://www.parkinson.org/Understanding-Parkinsons/Statistics

[4] Yang W, Hamilton JL, Kopil C, . Current and projected future economic burden of Parkinson’s disease in the U.S. NPJ Parkinsons Dis. 2020;6:15. doi:10.1038/s41531-020-0117-1 32665974 PMC7347582

[5] Power MC, Adar SD, Yanosky JD, Weuve J. Exposure to air pollution as a potential contributor to cognitive function, cognitive decline, brain imaging, and dementia: a systematic review of epidemiologic research. Neurotoxicology. 2016;56:235-253. doi:10.1016/j.neuro.2016.06.004 27328897 PMC5048530

[6] Ascherio A, Schwarzschild MA. The epidemiology of Parkinson’s disease: risk factors and prevention. Lancet Neurol. 2016;15(12):1257-1272. doi:10.1016/S1474-4422(16)30230-7 27751556

[7] Kasdagli MI, Katsouyanni K, Dimakopoulou K, Samoli E. Air pollution and Parkinson’s disease: a systematic review and meta-analysis up to 2018. Int J Hyg Environ Health. 2019;222(3):402-409. doi:10.1016/j.ijheh.2018.12.006 30606679

[8] Weuve J, Bennett EE, Ranker L, . Exposure to air pollution in relation to risk of dementia and related outcomes: an updated systematic review of the epidemiological literature. Environ Health Perspect. 2021;129(9):96001. doi:10.1289/EHP8716 34558969 PMC8462495

[9] Markevych I, Schoierer J, Hartig T, . Exploring pathways linking greenspace to health: theoretical and methodological guidance. Environ Res. 2017;158:301-317. doi:10.1016/j.envres.2017.06.028 28672128

[10] Fong KC, Hart JE, James P. A review of epidemiologic studies on greenness and health: updated literature through 2017. Curr Environ Health Rep. 2018;5(1):77-87. doi:10.1007/s40572-018-0179-y 29392643 PMC5878143

[11] de Keijzer C, Tonne C, Basagaña X, . Residential surrounding greenness and cognitive decline: a 10-year follow-up of the Whitehall II cohort. Environ Health Perspect. 2018;126(7):077003. doi:10.1289/EHP2875 30028296 PMC6108840

[12] James P, Hart JE, Banay RF, Laden F. Exposure to greenness and mortality in a nationwide prospective cohort study of women. Environ Health Perspect. 2016;124(9):1344-1352. doi:10.1289/ehp.1510363 27074702 PMC5010419

[13] Klompmaker JO, Janssen NAH, Bloemsma LD, . Effects of exposure to surrounding green, air pollution and traffic noise with non-accidental and cause-specific mortality in the Dutch national cohort. Environ Health. 2021;20(1):82. doi:10.1186/s12940-021-00769-0 34261495 PMC8281461

[14] Orioli R, Antonucci C, Scortichini M, . Exposure to residential greenness as a predictor of cause-specific mortality and stroke incidence in the Rome Longitudinal Study. Environ Health Perspect. 2019;127(2):27002. doi:10.1289/EHP2854 30775931 PMC6752936

[15] Yuchi W, Sbihi H, Davies H, Tamburic L, Brauer M. Road proximity, air pollution, noise, green space and neurologic disease incidence: a population-based cohort study. Environ Health. 2020;19(1):8. doi:10.1186/s12940-020-0565-4 31964412 PMC6974975

[16] Yu Z, Wei F, Zhang X, . Air pollution, surrounding green, road proximity and Parkinson’s disease: a prospective cohort study. Environ Res. 2021;197:111170. doi:10.1016/j.envres.2021.111170 33887274

[17] Slawsky ED, Hajat A, Rhew IC, . Neighborhood greenspace exposure as a protective factor in dementia risk among U.S. adults 75 years or older: a cohort study. Environ Health. 2022;21(1):14. doi:10.1186/s12940-022-00830-6 35033073 PMC8760791

[18] Koay L, Rose J, Abdelhafiz AH. Factors that lead to hospitalisation in patients with Parkinson disease—a systematic review. Int J Clin Pract. 2018;72(1):e13039. doi:10.1111/ijcp.13039 29119656

[19] Shi L, Wu X, Danesh Yazdi M, . Long-term effects of PM_2.5_ on neurological disorders in the American Medicare population: a longitudinal cohort study. Lancet Planet Health. 2020;4(12):e557-e565. doi:10.1016/S2542-5196(20)30227-8 33091388 PMC7720425

[20] Chronic Conditions Data Warehouse. Condition Categories. Accessed February 22, 2022. https://www2.ccwdata.org/web/guest/condition-categories

[21] National Aeronautics and Space Administration. Measuring Vegetation (NDVI and EVI). Accessed June 16, 2020. https://earthobservatory.nasa.gov/features/MeasuringVegetation/measuring_vegetation_2.php

[22] Pekel JF, Cottam A, Gorelick N, Belward AS. High-resolution mapping of global surface water and its long-term changes. Nature. 2016;540(7633):418-422. doi:10.1038/nature20584 27926733

[23] Filice CE, Joynt KE. Examining race and ethnicity information in Medicare administrative data. Med Care. 2017;55(12):e170-e176. doi:10.1097/MLR.0000000000000608 29135782

[24] Koller D, Bynum JPW. Dementia in the USA: state variation in prevalence. J Public Health (Oxf). 2015;37(4):597-604. 25330771 10.1093/pubmed/fdu080 PMC6283394

[25] Mantri S, Fullard ME, Beck J, Willis AW. State-level prevalence, health service use, and spending vary widely among Medicare beneficiaries with Parkinson disease. NPJ Parkinsons Dis. 2019;5:1. doi:10.1038/s41531-019-0074-8 30701188 PMC6345811

[26] Adkins-Jackson PB, Chantarat T, Bailey ZD, Ponce NA. Measuring structural racism: a guide for epidemiologists and other health researchers. Am J Epidemiol. 2022;191(4):539-547. doi:10.1093/aje/kwab239 34564723 PMC9077112

[27] Blondell SJ, Hammersley-Mather R, Veerman JL. Does physical activity prevent cognitive decline and dementia?: a systematic review and meta-analysis of longitudinal studies. BMC Public Health. 2014;14:510. doi:10.1186/1471-2458-14-510 24885250 PMC4064273

[28] Seeman TE, Miller-Martinez DM, Stein Merkin S, Lachman ME, Tun PA, Karlamangla AS. Histories of social engagement and adult cognition: midlife in the U.S. study. J Gerontol B Psychol Sci Soc Sci. 2011;66(suppl 1):i141-i152. doi:10.1093/geronb/gbq091 21196438 PMC3132769

[29] Ertel KA, Glymour MM, Berkman LF. Effects of social integration on preserving memory function in a nationally representative US elderly population. Am J Public Health. 2008;98(7):1215-1220. doi:10.2105/AJPH.2007.113654 18511736 PMC2424091

[30] Fang X, Han D, Cheng Q, . Association of levels of physical activity with risk of Parkinson disease: a systematic review and meta-analysis. JAMA Netw Open. 2018;1(5):e182421. doi:10.1001/jamanetworkopen.2018.2421 30646166 PMC6324511

[31] Banay RF, James P, Hart JE, . Greenness and depression incidence among older women. Environ Health Perspect. 2019;127(2):27001. doi:10.1289/EHP1229 30735068 PMC6752939

[32] Sarkar C, Webster C, Gallacher J. Residential greenness and prevalence of major depressive disorders: a cross-sectional, observational, associational study of 94 879 adult UK Biobank participants. Lancet Planet Health. 2018;2(4):e162-e173. doi:10.1016/S2542-5196(18)30051-2 29615217

[33] Zhang X, Wei F, Yu Z, . Association of residential greenness and incident depression: investigating the mediation and interaction effects of particulate matter. Sci Total Environ. 2022;811:152372. doi:10.1016/j.scitotenv.2021.152372 34914979

[34] Ownby RL, Crocco E, Acevedo A, John V, Loewenstein D. Depression and risk for Alzheimer disease: systematic review, meta-analysis, and metaregression analysis. Arch Gen Psychiatry. 2006;63(5):530-538. doi:10.1001/archpsyc.63.5.530 16651510 PMC3530614

[35] Wang S, Mao S, Xiang D, Fang C. Association between depression and the subsequent risk of Parkinson’s disease: a meta-analysis. Prog Neuropsychopharmacol Biol Psychiatry. 2018;86:186-192. doi:10.1016/j.pnpbp.2018.05.025 29859854

[36] Chen H, Burnett RT, Bai L, . Residential greenness and cardiovascular disease incidence, readmission, and mortality. Environ Health Perspect. 2020;128(8):87005. doi:10.1289/EHP6161 32840393 PMC7446772

[37] Yang BY, Markevych I, Heinrich J, . Associations of greenness with diabetes mellitus and glucose-homeostasis markers: the 33 Communities Chinese Health Study. Int J Hyg Environ Health. 2019;222(2):283-290. doi:10.1016/j.ijheh.2018.12.001 30545606

[38] Villeneuve PJ, Jerrett M, Su JG, Weichenthal S, Sandler DP. Association of residential greenness with obesity and physical activity in a US cohort of women. Environ Res. 2018;160:372-384. doi:10.1016/j.envres.2017.10.005 29059619 PMC5872815

[39] Baumgart M, Snyder HM, Carrillo MC, Fazio S, Kim H, Johns H. Summary of the evidence on modifiable risk factors for cognitive decline and dementia: a population-based perspective. Alzheimers Dement. 2015;11(6):718-726. doi:10.1016/j.jalz.2015.05.016 26045020

[40] Browning MHEM, Rigolon A, McAnirlin O, Yoon HV. Where greenspace matters most: a systematic review of urbanicity, greenspace, and physical health. Landsc Urban Plan. 2022;217:104233. doi:10.1016/j.landurbplan.2021.104233

[41] Rigolon A, Browning MHEM, McAnirlin O, Yoon HV. Green space and health equity: a systematic review on the potential of green space to reduce health disparities. Int J Environ Res Public Health. 2021;18(5):1-29. doi:10.3390/ijerph18052563 33806546 PMC7967323

[42] Homer C, Dewitz J, Jin S, . Conterminous United States land cover change patterns 2001-2016 from the 2016 National Land Cover Database. ISPRS J Photogramm Remote Sens. 2020;162:184-199. doi:10.1016/j.isprsjprs.2020.02.019 35746921 PMC9214659

