# Supplementary material for: Associations of Greenness, Parks, and Blue Space With Neurodegenerative Disease Hospitalizations Among Older US Adults
Source: JAMA Netw Open. 2022 Dec 20;5(12):e2247664. doi: 10.1001/jamanetworkopen.2022.47664 (PMC9856892; doi:10.1001/jamanetworkopen.2022.47664)
Supplement: Supplement 1. — eMethods. eReferences eTable 1. Descriptive Statistics of the Alzheimer Disease and Related Dementias and Parkinson Disease Hospitalization Cohorts, 2000 to 2016 eTable 2. Descriptive Statistics of Normalized Difference Vegetation Index, Percentage Park Cover, and Percentage Blue Space Cover in the Alzheimer Disease and Related Dementias and Parkinson Disease Hospitalization Cohorts (Aggregated Data), 2000 to 2016 eTable 3. Sensitivity Analyses of Normalized Difference Vegetation Index, Percentage Park Cover, and Blue Space Cover (≥1.0%) With Alzheimer Disease and Related Dementias and Parkinson Disease Hospitalization eTable 4. Sensitivity Analyses of Percentage Blue Space Cover Cutoffs With Alzheimer Disease and Related Dementias and Parkinson Disease Hospitalization eTable 5. Total Number of Alzheimer Disease and Related Dementias and Parkinson Disease Hospital Admissions Avoided in 1 Year eFigure 1. Spatial Variation of Zip Code−Level Normalized Difference Vegetation Index (2008), Percentage Park Cover, and Percentage Blue Space Cover in the Contiguous US eFigure 2. US Census Regions and US Census Divisions eFigure 3. Maps of Alzheimer Disease and Related Dementias and Parkinson Disease Hospitalizations per 100 000 Person-Years, 2000 to 2016 eFigure 4. Spearman Correlations Among Normalized Difference Vegetation Index, Percentage Park Cover, Percentage Blue Space Cover, and Other Covariates eFigure 5. Exposure-Response Curves (1, 2, or 3 Degrees of Freedom) of Normalized Difference Vegetation Index and Percentage Park Cover With Alzheimer Disease and Related Dementias and Parkinson Disease Hospitalization eFigure 6. Associations of Normalized Difference Vegetation Index, Percentage Park Cover, and Blue Space Cover (≥1.0%) With Alzheimer Disease and Related Dementias and Parkinson Disease Hospitalization in Models With Increasing Adjustment for Potential Confounders eFigure 7. Associations of Normalized Difference Vegetation Index, Percentage Park Cover, and Blue [file jamanetwopen-e2247664-s001.pdf]

Supplementary Online Content

Klompmaker JO, Laden F, Browning MHEM, et al. Associations of greenness, parks, and blue space with neurodegenerative disease hospitalizations among older US adults. *JAMA Netw Open*. 2022;5(12):e2247664. doi:10.1001/jamanetworkopen.2022.47664

eMethods.
eReferences
eTable 1. Descriptive Statistics of the Alzheimer Disease and Related Dementias and Parkinson Disease Hospitalization Cohorts, 2000 to 2016
eTable 2. Descriptive Statistics of Normalized Difference Vegetation Index, Percent Park Cover, and Percent Blue Space Cover in the Alzheimer Disease and Related Dementias and Parkinson Disease Hospitalization Cohorts (Aggregated Data), 2000 to 2016
eTable 3. Sensitivity Analyses of Normalized Difference Vegetation Index, Percent Park Cover, and Blue Space Cover (≥1.0%) With Alzheimer Disease and Related Dementias and Parkinson Disease Hospitalization
eTable 4. Sensitivity Analyses of Percent Blue Space Cover Cutoffs With Alzheimer Disease and Related Dementias and Parkinson Disease Hospitalization
eTable 5. Total Number of Alzheimer Disease and Related Dementias and Parkinson Disease Hospital Admissions Avoided in 1 Year
eFigure 1. Spatial Variation of Zip Code−Level Normalized Difference Vegetation Index (2008), Percent Park Cover, and Percent Blue Space Cover in the Contiguous US
eFigure 2. US Census Regions and US Census Divisions
eFigure 3. Maps of Alzheimer Disease and Related Dementias and Parkinson Disease Hospitalizations per 100,000 Person-Years, 2000 to 2016
eFigure 4. Spearman Correlations Among Normalized Difference Vegetation Index, Percent Park Cover, Percent Blue Space Cover, and Other Covariates
eFigure 5. Exposure-Response Curves (1, 2, or 3 Degrees of Freedom) of Normalized Difference Vegetation Index and Percent Park Cover With Alzheimer Disease and Related Dementias and Parkinson Disease Hospitalization
eFigure 6. Associations of Normalized Difference Vegetation Index, Percent Park Cover, and Blue Space Cover (≥1.0%) With Alzheimer Disease and Related Dementias and Parkinson Disease Hospitalization in Models With Increasing Adjustment for Potential Confounders
eFigure 7. Associations of Normalized Difference Vegetation Index, Percent Park Cover, and Blue Space Cover (≥1.0%) With Alzheimer Disease and Related Dementias and Parkinson Disease Hospitalization in the Urban Population in Stratified Analyses
eFigure 8. Associations of Normalized Difference Vegetation Index, Percent Park Cover, and Blue Space Cover (≥1.0%) With Alzheimer Disease and Related Dementias and Parkinson Disease Hospitalization in the Urban Population in Stratified Analyses by Neighborhood Socioeconomic Status

This supplementary material has been provided by the authors to give readers additional information about their work.

eMethods

1. Outcome definition

We used the following ICD-9 (2000 - third quarter of 2015) and ICD-10 (fourth quarter of 2015 - 2016) to define ADRD and PD hospitalization:

ADRD hospitalization ICD-9: 331.0, 331.11, 331.19, 331.2, 331.7, 290.0, 290.10, 290.11, 290.12, 290.13, 290.20, 290.21, 290.3, 290.40, 290.41, 290.42, 290.43, 294.0, 294.10, 294.11, 294.20, 294.21, 294.8 and 797; ICD-10: F01.50, F01.51, F02.80, F02.81, F03.90, F03.91, F04, F05, F06.1, F06.8, G13.8, G30.0, G30.1, G30.8, G30.9, G31.01, G31.09, G31.1, G31.2, G94, R41.81 and R54.

PD hospitalization: ICD-9: 332.0 332.1, ICD-10: G20, G21.11, G21.19, and G21.8.

2. Exposure assessment

As we only had information about the residential zip code of each beneficiary, we calculated zip code-level exposures.

Greenness

We used the Normalized Difference Vegetation Index (NDVI) as an indicator of greenness. The NDVI is calculated as the ratio between the red and near infrared values.[1] NDVI ranges from -1 to 1 with larger values indicating higher levels of live vegetation, negative values correspond to water. The NDVI is estimated with satellite images from Landsat 7 and Landsat 8 (Collection 1 Tier 1 DN values, representing scaled, calibrated at sensor radiance). Landsat 7 and 8 images are generated every 16 days at 30m resolution. We used images from June 1, up to August 31, for each year (2000-2016) to maximize variability. Using Google Earth Engine,[2] we made cloud-free composite NDVI maps for the contiguous US. We calculated the spatially weighted mean summer NDVI for each zip code in the US for each year, after setting negative NDVI values to zero.

Park cover

Park cover is based on cross-sectional data from the USGS Protected Areas Database of the US (PAD-US) V2.1 (2020). The database strives to be a complete inventory of public land and other protected areas in the US by compiling the "best available" data provided by land managing agencies and organizations.[3] PAD-US differentiates between

© 2022 Klompmaker JO et al. *JAMA Network Open*

multiple types of public lands. Therefore, we selected all land types that are likely to be known and used by the general public for outdoor recreation from PAD-US to create a park cover dataset.

The following designations were included in the park cover dataset:

1. Parks and open spaces open for public access or restricted access (i.e., seasonally open, fees required, or permits required) including but not limited to lands managed by the National Park Service, U.S. Forest Service, Bureau of Land Management, U.S. Fish & Wildlife, Army Corps of Engineers, State Parks, State Departments of Conservation, State Departments of Natural Resources, State Departments of Land, State Fish and Wildlife Departments, State Forest Service, State Park and Recreation Departments, Tennessee Valley Authority, and city and county park and recreation departments
2. Conservation easements

The following designations were excluded from the park cover dataset:

1. Department of Energy, Department of Defense, and Bureau of Reclamation lands
2. Marine areas managed as/by Marine Protected Areas, National Oceanic and Atmospheric Administration, Bureau of Ocean Energy Management, etc.
3. Proclamation areas, which are boundaries of national lands used for administrative purposes that overlap with large areas of public lands that are not all available to the public
4. Fish hatcheries and other lands used for water rights with regulated hunting
5. National Park easements (i.e., lands paralleling but not including the Appalachian Trail and not used by the public)
6. Joint management areas (i.e., university research stations)
7. Non-governmental organization lands (aside from conservation easements)
8. State trust/land survey lands
9. American Indian Lands
10. Other areas with unknown access or closed public access (i.e., limited to coordinated programs and research)

© 2022 Klompmaker JO et al. *JAMA Network Open*

To assess zip code-level park cover, we converted the park dataset to a raster image and calculated percent park cover for each zip code. More information about this dataset and a link to download the data can be found elsewhere.[4,5]

Blue space

Blue space was based on the Joint Research Centre's Global Surface Water Dataset.[6] This dataset contains information about the location of surface water, based on satellite images from Landsat 5, 7, and 8 from 1984 to 2018 (30 m spatial resolution). To classify each pixel into water or non-water, we used the "Occurrence" band (the frequency with which water was present from 1984-2018) with a 50% threshold. As zip codes are used for postal services, adjacent water bodies such as rivers, lakes and oceans are not always included in zip code areas. Therefore, we calculated spatially weighted mean blue space of zip codes with a 1000 m buffer to be able to capture water bodies close to each zip code. We used Google Earth Engine to calculate spatially weighted mean blue space for each zip code. Given the limited variability of the distribution of percent blue space cover, we used a binary blue space cover indicator (dichotomized at 1%). To determine the sensitivity of our results to this cutpoint, we also evaluated 5% as cutpoint.

3. Covariates

For each beneficiary, we obtained data on age, sex, race, Medicaid eligibility (a proxy for low socioeconomic status (SES)), year of entry, and zip code of residence for all Medicare beneficiaries from the Medicare beneficiary file. Medicaid provides health coverage for adults below a certain income level. From the US Census and American Community Survey we derived eight zip code-level SES covariates: median home value, median household income, population density, percent Hispanic, percent Black, percent of the population with less than a high school degree, percent below the poverty level, and percent of owner-occupied housing units. We also linked county-level percent of the population that were ever smokers from the nationwide Behavioural Risk Factor Surveillance System (BRFSS). Zip code-level SES covariates (2000, 2009-2016) and percent of the population that were ever smokers (2000-2011) were not available for all years. We used a moving average algorithm to temporarily interpolate data for missing years, as described previously.[7] Further, we divided the US into four census regions (West, Midwest, Northeast, South) and nine divisions (Pacific, Mountain, West North Central, East North Central, Middle Atlantic, New England, South Atlantic, East South Central, West South Central), to adjust for regional differences not adjusted for by the other variables. Previous studies documented regional differences in ADRD and PAR prevalence that may be related to differences in recognition of symptoms and willingness to document the disease.[8,9]

We also linked zip code-level annual average particulate matter less than 2.5 microns ($PM_{2.5}$) and nitrogen dioxide (NO_2), and summer average maximum daily temperature, specific humidity and total precipitation to each beneficiary. Briefly, annual $PM_{2.5}$ and NO_2 concentrations across the contiguous US for 2000-2016 were estimated based on predictions from well-validated spatio-temporal ensemble models.[10–12] Information about the development of air pollution models and predictions can be found elsewhere.[10–12] For each zip code, the annual average concentrations were estimated by averaging the estimations at grid cells whose centroids fall within the boundary of that ZIP code. Using daily data from the Gridded Surface Meteorological dataset, we estimated the average summer maximum temperature, ambient specific humidity and total precipitation for each zip code for each year.[13]

4. Hospitalization admissions avoided

We estimated the total number of ADRD and PD hospital admissions avoided to demonstrate the potential health impact natural environments could have, assuming that modelling assumptions are correct and our observed associations are true. To evaluate the public health burden associated with environmental exposures, Health Impact Assessments (HIA) are generally used.[14] We estimated the total number of hospital admissions avoided among this population in one year if, hypothetically, zip code NDVI and park cover increased by 0.5 IQR, and blue space cover increased to ≥1 % in all zip codes in the US. We calculated the average number of beneficiaries and ADRD and PD hospital admissions in this cohort in one year. Because we estimated the total number of hospital admissions avoided if blue space cover would be ≥1 % in all zip codes in the US, we also calculated the average number of beneficiaries and ADRD and PD hospital admissions in zip codes with blue space cover <1%. We re-calculated the HRs from the main model per 0.5 IQR increase to quantify the association between NDVI and park cover and ADRD and PD hospital admissions. Next, we estimated the population-attributable fraction (PAF). The PAF is the proportion reduction in the hospital admissions that would occur if the exposure was increased and is calculated as: PAF = (HR-1)/HR.[14] We estimated the number of hospitalization admissions avoided if the exposure increases by multiplying the PAF with the average number of ADRD or PD hospital admissions in the population. We used the 95% CIs from the HR estimates to calculate the 95% CI for the hospital admissions avoided.

Health impact assessment has inherent uncertainties and requires a set of assumptions, as described elsewhere.[14] We believe that the assumptions for this assessment are reasonable.

© 2022 Klompmaker JO et al. *JAMA Network Open*

eReferences

1. NASA. Measuring Vegetation (NDVI & EVI). Accessed June 16, 2020. https://earthobservatory.nasa.gov/features/MeasuringVegetation/measuring_vegetation_2.php

2. Gorelick N, Hancher M, Dixon M, Ilyushchenko S, Thau D, Moore R. Google Earth Engine: Planetary-scale geospatial analysis for everyone. *Remote Sens Environ*. 2017;202:18-27. doi:10.1016/j.rse.2017.06.031

3. U.S. Geological Survey (USGS), Gap Analysis Project. Protected Areas Database of the United States (PAD-US) 2.1: U.S. Geological Survey data release. doi:https://doi.org/10.5066/P92QM3NT.

4. Browning MHEM, Rigolon A, Ogletree S, et al. Curation of new green space indicator for the U.S.: Accessible & recreational park cover (PAD-US-AR). *EcoEvoRxiv*. Published online 2022. doi:10.32942/OSF.IO/PYDQE

5. Ogletree S, Browning MHEM. OSF | PADUS-AR. doi:10.17605/OSF.IO/PWDSG

6. Pekel JF, Cottam A, Gorelick N, Belward AS. High-resolution mapping of global surface water and its long-term changes. *Nature*. 2016;540(7633):418-422. doi:10.1038/nature20584

7. Di Q, Wang Y, Zanobetti A, et al. Air Pollution and Mortality in the Medicare Population. *N Engl J Med*. 2017;376(26):2513-2522. doi:10.1056/NEJMoa1702747

8. Koller D, Bynum JPW. Dementia in the USA: state variation in prevalence. *J Public Health (Bangkok)*. 2015;37(4):597-604. doi:10.1093/PUBMED/FDU080

9. Mantri S, Fullard ME, Beck J, Willis AW. State-level prevalence, health service use, and spending vary widely among Medicare beneficiaries with Parkinson disease. *npj Park Dis 2019 51*. 2019;5(1):1-9. doi:10.1038/s41531-019-0074-8

© 2022 Klompmaker JO et al. *JAMA Network Open*

10. Requia WJ, Di Q, Silvern RF, et al. An ensemble learning approach for estimating high spatiotemporal resolution of ground-level ozone in the contiguous United States. *Environ Sci Technol*. 2020;54:11037-11047. doi:10.1021/acs.est.0c01791

11. Di Q, Amini H, Shi L, et al. An ensemble-based model of PM2.5 concentration across the contiguous United States with high spatiotemporal resolution. *Environ Int*. 2019;130:104909. doi:10.1016/j.envint.2019.104909

12. Di Q, Amini H, Shi L, et al. Assessing NO 2 Concentration and Model Uncertainty with High Spatiotemporal Resolution across the Contiguous United States Using Ensemble Model Averaging. *ACS Publ*. 2019;54(3):1372-1384. doi:10.1021/acs.est.9b03358

13. Abatzoglou JT. Development of gridded surface meteorological data for ecological applications and modelling. *Int J Climatol*. 2013;33(1):121-131. doi:10.1002/joc.3413

14. WHO Working Group. Evaluation and Use of Epidemiological Evidence for Environmental Health Risk Assessment: WHO Guideline Document. *Environ Health Perspect*. 2000;108(10):997. doi:10.2307/3435062

eTable 1. Descriptive Statistics of the Alzheimer Disease and Related Dementias and Parkinson Disease Hospitalization Cohorts, 2000 to 2016[a,b,c]

	ADRD hospitalization		PD hospitalization	
**Covariate**	**Full cohort**	**Urban cohort**	**Full cohort**	**Urban cohort**
	**N (%) / Median (25th-75th pctl)**	**N (%) / Median (25th-75th pctl)**	**N (%) / Median (25th-75th pctl)**	**N (%) / Median (25th-75th pctl)**
**Sex**				
-female	34,010,915 (55.2)	18,103,358 (56.4)	34,018,632 (55.2)	18,197,246 (56.4)
-male	27,651,557 (44.8)	14,007,039 (43.6)	27,654,735 (44.8)	14,043,833 (43.6)
**Age entry**				
65-74 years	47,237,464 (76.6)	24,200,060 (75.4)	47,242,227 (76.6)	24,256,348 (75.2)
75-84 years	10,583,080 (17.2)	5,834,900 (18.2)	10,587,668 (17.2)	5,892,433 (18.3)
85+ years	3,841,928 (6.2)	2,075,437 (6.5)	3,843,472 (6.2)	2,092,298 (6.5)
**Race**				
White	52,044,489 (84.4)	25,175,889 (78.4)	52,053,754 (84.4)	25,289,478 (78.4)
Black	5,421,282 (8.8)	3,744,203 (11.7)	5,422,604 (8.8)	3,757,988 (11.7)
Other/unknown	4,196,701 (6.8)	3,190,305 (9.9)	4,197,009 (6.8)	3,193,613 (9.9)
**Medicaid eligibility**				
Not eligible	53,991,858 (87.6)	27,559,016 (85.8)	53,998,297 (87.6)	27,646,825 (85.8)
Eligible	7,670,614 (12.4)	4,551,381 (14.2)	7,675,070 (12.4)	4,594,254 (14.2)
**Region**				
Northeast	12,354,729 (20.0)	7,864,622 (24.5)	12,355,117 (20)	7,886,655 (24.5)
South	23,483,516 (38.1)	10,511,747 (32.7)	23,489,372 (38.1)	10,572,614 (32.8)
Midwest	14,656,222 (23.8)	6,869,910 (21.4)	14,659,632 (23.8)	6,897,199 (21.4)
West	11,168,005 (18.1)	6,864,118 (21.4)	11,169,246 (18.1)	6,884,611 (21.4)
**Aggregated data (2000-2016)**				
**Natural environment measures**				
- NDVI	0.52 (0.36-0.63)	0.39 (0.27-0.50)	0.52 (0.36-0.63)	0.39 (0.27-0.50)
- % Park cover	7.8 (2.2-18.0)	11.4 (5.9-19.6)	7.8 (2.2-17.9)	11.4 (5.9-19.6)
- % Blue space cover (1000m buffer)	0.5 (0.1-3.4)	0.7 (0.1-5.3)	0.5 (0.1-3.4)	0.7 (0.1-5.3)
-% of aggregated data with ≥1 % blue space cover	41.4	45.1	41.5	45.2
-% of aggregated data with ≥5 % blue space cover	20.5	25.5	20.5	25.5
**US census covariates**				
- Population density (persons/mile^2)	608.2 (84.3-3096.3)	3583.9 (2103.9-6330)	619.1 (85.5-3105.2)	3583.9 (2103.9-6330.0)
- Median home value ($1,000)	144.0 (92.2-239.5)	197.9 (123.3-333.1)	144.4 (92.5-240.1)	197.9 (123.3-333.1)
- Median household income ($1,000)	46.7 (36.6-61.9)	51.6 (38.4-69.8)	46.8 (36.6-62.0)	51.6 (38.4-69.8)
- % with less than a high school degree	24.3 (15.1-36.4)	22.6 (13.8-34.8)	24.3 (15.1-36.3)	22.6 (13.8-34.8)
- % below the poverty level	8.5 (5.4-13.4)	8.6 (5.6-13.9)	8.5 (5.4-13.4)	8.6 (5.6-13.9)

© 2022 Klompmaker JO et al. *JAMA Network Open*

- % owner-occupied housing units	71.5 (58.8-80.6)	60.1 (46.4-72.4)	71.4 (58.7-80.5)	60.1 (46.4-72.4)
- % Black	3.9 (0.8-14.5)	7.3 (2.8-20.7)	4.0 (0.8-14.6)	7.3 (2.8-20.7)
- % Hispanic	5.3 (1.8-16.0)	11.3 (4.7-27.4)	5.3 (1.8-16)	11.3 (4.7-27.4)
**BRFSS covariate**				
- % ever smoked	46.2 (41.5-50.5)	44.3 (40.1-48.1)	46.2 (41.4-50.5)	44.3 (40.1-48.1)
**Other environmental exposures**				
-summer temperature (°C)	29.8 (27.3-32.5)	29.6 (27.4-32.2)	29.9 (27.3-32.5)	29.6 (27.4-32.2)
-summer specific humidity (g of water vapor / kg of dry air)	12.0 (10.4-14.4)	11.7 (10.4-13.6)	12.0 (10.4-14.4)	11.7 (10.4-13.6)
-summer total precipitation (mm, daily total)	3.1 (1.9-4.2)	3.0 (1.1-4.3)	3.1 (1.9-4.2)	3.0 (1.1-4.3)
-PM2.5 (µg/m^3)	9.7 (7.8-11.8)	10.1 (8.4-12.3)	9.7 (7.8-11.7)	10.1 (8.4-12.3)
-NO2 (ppb)	16.6 (10.9-24.8)	24.9 (18.8-31.8)	16.6 (10.9-24.8)	24.9 (18.8-31.8)

[a] Descriptive statistics for the natural environment measures, US census covariates, BRFSS covariate, and other environmental exposures are given for the strata (aggregated data based on zip code, year, sex, race, Medicaid eligibility, 2-year categories of age at study entry and year of follow-up).

[b] Urban cohorts included all person years in zip codes with a population density of 1000+ persons/mile^2.

[c] The percentage of additional missings (compared to complete case analyses based on all covariates included in the main model) was very low for summer temperature, specific humidity and precipitation (<0.1%) and for PM2.5 and ozone (<0.2%).

© 2022 Klompmaker JO et al. *JAMA Network Open*

eTable 2. Descriptive Statistics of Normalized Difference Vegetation Index, Percent Park Cover, and Percent Blue Space Cover in the Alzheimer Disease and Related Dementias and Parkinson Disease Hospitalization Cohorts (Aggregated Data), 2000 to 2016[a,b]

Exposure	Mean	Standard deviation	Min	5th percentile	25th percentile	median	75th percentile	95th percentile	Max	Interquartile range
	**ADRD full cohort**									
**NDVI**	0.49	0.17	0.0	0.17	0.36	0.52	0.63	0.72	0.84	0.27
**% Park cover**	13.3	16.1	0.0	0.0	2.2	7.8	18.0	47.0	100.0	15.8
**% Blue space cover (1000m buffer)**	4.2	9.0	0.0	0.0	0.1	0.5	3.4	22.7	90.9	3.3
	**ADRD urban cohort**									
**NDVI**	0.38	0.15	0.0	0.14	0.27	0.39	0.5	0.61	0.81	0.23
**% Park cover**	14.6	12.1	0.0	2.0	5.8	11.4	19.6	38.4	99.9	13.8
**% Blue space cover (1000m buffer)**	5.5	10.5	0.0	0.0	0.1	0.7	5.3	28.1	88.0	5.2
	**PD full cohort**									
**NDVI**	0.49	0.17	0.0	0.17	0.36	0.52	0.63	0.72	0.84	0.27
**% Park cover**	13.2	16.1	0.0	0.0	2.2	7.8	17.9	46.9	100.0	15.8
**% Blue space cover (1000m buffer)**	4.2	9.0	0.0	0.0	0.1	0.5	3.4	22.8	90.9	3.3
	**PD urban cohort**									
**NDVI**	0.38	0.15	0.0	0.14	0.27	0.39	0.5	0.61	0.81	0.23
**% Park cover**	14.6	12.1	0.0	2.0	5.9	11.4	19.6	38.4	99.9	13.8
**% Blue space cover (1000m buffer)**	5.5	10.5	0.0	0.0	0.1	0.7	5.3	28.1	88.0	5.2

[a] Descriptive statistics are given for the strata (aggregated data based on zip code, year, sex, race, Medicaid eligibility, 2-year categories of age at study entry and year of follow-up).

[b] Urban cohorts included all person years in zip codes with a population density of 1000+ persons/mile^2.

© 2022 Klompmaker JO et al. *JAMA Network Open*

eTable 3. Sensitivity Analyses of Normalized Difference Vegetation Index, Percent Park Cover, and Blue Space Cover (≥1.0%) With Alzheimer Disease and Related Dementias and Parkinson Disease Hospitalization[a,b]

Exposure	main	Single-exposure	Adj. for $PM_{2.5}$ + NO_2	Adj. for temp + humid + precip	Adj. for division instead of region	Excl. potentially prevalent cases
	HR (95% CI)	HR (95% CI)	HR (95% CI)	HR (95% CI)	HR (95% CI)	HR (95% CI)
	**ADRD - Full**					
**NDVI**	0.95 (0.94, 0.96)	0.95 (0.94, 0.96)	0.97 (0.96, 0.97)	0.97 (0.96, 0.98)	0.95 (0.94, 0.96)	0.95 (0.94, 0.96)
**% Park cover**	0.99 (0.99, 0.99)	0.99 (0.99, 0.99)	1.00 (1.00, 1.01)	1.00 (1.00, 1.00)	0.99 (0.99, 1.00)	0.99 (0.99, 0.99)
**≥1 % Blue space cover**	0.99 (0.99, 1.00)	0.99 (0.99, 1.00)	1.01 (1.00, 1.01)	0.99 (0.98, 1.00)	1.00 (0.99, 1.01)	0.99 (0.99, 1.00)
	**ADRD - Urban**					
**NDVI**	0.99 (0.98, 1.01)	0.99 (0.98, 1.01)	0.99 (0.98, 1.01)	1.01 (0.99, 1.02)	1.01 (0.99, 1.02)	0.99 (0.98, 1.01)
**% Park cover**	0.99 (0.98, 1.00)	0.99 (0.98, 1.00)	0.99 (0.99, 1.00)	1.00 (0.99, 1.00)	0.99 (0.99, 1.00)	0.99 (0.98, 1.00)
**≥1 % Blue space cover**	1.00 (0.99, 1.01)	1.00 (0.99, 1.01)	1.01 (1.00, 1.02)	1.00 (0.99, 1.01)	1.02 (1.00, 1.03)	1.00 (0.99, 1.01)
	**PD - Full**					
**NDVI**	0.94 (0.93, 0.95)	0.95 (0.94, 0.96)	0.96 (0.95, 0.97)	0.97 (0.96, 0.98)	0.95 (0.94, 0.96)	0.94 (0.93, 0.95)
**% Park cover**	0.97 (0.97, 0.98)	0.97 (0.97, 0.98)	0.99 (0.98, 0.99)	0.99 (0.98, 0.99)	0.98 (0.97, 0.98)	0.97 (0.97, 0.98)
**≥1 % Blue space cover**	0.97 (0.96, 0.98)	0.97 (0.96, 0.98)	0.98 (0.98, 0.99)	0.97 (0.96, 0.97)	0.98 (0.97, 0.99)	0.97 (0.96, 0.98)
	**PD - Urban**					
**NDVI**	0.95 (0.93, 0.97)	0.95 (0.93, 0.96)	0.96 (0.94, 0.97)	0.97 (0.95, 0.99)	0.97 (0.95, 0.98)	0.95 (0.93, 0.97)
**% Park cover**	0.99 (0.98, 1.00)	0.98 (0.97, 0.99)	0.99 (0.98, 1.00)	0.99 (0.98, 1.00)	0.99 (0.98, 1.00)	0.99 (0.98, 1.00)
**≥1 % Blue space cover**	0.98 (0.97, 1.00)	0.98 (0.97, 0.99)	0.99 (0.98, 1.01)	0.98 (0.96, 0.99)	1.00 (0.98, 1.01)	0.99 (0.97, 1.00)

[a] Associations of NDVI (0.27) and percent park cover (15.9) are expressed per IQR increase of the ADRD hospitalization (full) cohort, associations of ≥1 % blue space cover are given compared to the reference category (<1 % blue space cover). Models included percent park cover, NDVI, percent blue space cover and were adjusted for calendar year, region, US census covariates, % ever smoked, , an offset for total person-time and strata for all possible combinations of sex, race, Medicaid Eligibility, age at study entry (2-year categories), and follow-up year. Urban cohorts included all person years in zip codes with a population density of 1000+ persons/mile^2.

[b] Single-exposure = model including only a single exposure. Adj. for $PM_{2.5}$ + NO_2 = main model additionally adjusted for PM2.5 and NO_2. Adj. for temp + humid + precip = main model additionally adjusted for summer temperature, summer specific humidity, and summer total precipitation. Adj. division = main model adjusted for division instead of region. Excl. potentially prevalent cases = exclusion of individuals who had their first hospital admission within the first year of their follow-up and all records in the year 2000.

© 2022 Klompmaker JO et al. *JAMA Network Open*

eTable 4. Sensitivity Analyses of Percent Blue Space Cover Cutoffs With Alzheimer Disease and Related Dementias and Parkinson Disease Hospitalization[a]

Exposure	main	> 5% blue space cover
	HR (95% CI)	HR (95% CI)
	ADRD - Full	
NDVI	0.95 (0.94, 0.96)	0.95 (0.94, 0.96)
% Park cover	0.99 (0.99, 1.00)	0.99 (0.99, 1.00)
% Blue space cover	0.99 (0.99, 1.00)	0.99 (0.98, 1.00)
	ADRD - Urban	
NDVI	0.99 (0.98, 1.01)	1.00 (0.98, 1.01)
% Park cover	0.99 (0.98, 1.00)	0.99 (0.98, 1.00)
% Blue space cover	1.00 (0.99, 1.01)	1.01 (1.00, 1.02)
	PD - Full	
NDVI	0.94 (0.93, 0.95)	0.94 (0.93, 0.95)
% Park cover	0.97 (0.97, 0.98)	0.97 (0.97, 0.98)
% Blue space cover	0.97 (0.96, 0.98)	0.96 (0.95, 0.97)
	PD - Urban	
NDVI	0.95 (0.93, 0.97)	0.95 (0.93, 0.96)
% Park cover	0.99 (0.98, 1.00)	0.99 (0.98, 0.99)
% Blue space cover	0.98 (0.97, 1.00)	0.99 (0.97, 1.00)

[a] Associations of NDVI (0.27) and percent park cover (15.9) are expressed per IQR increase of the ADRD hospitalization (full) cohort, associations of ≥1 % blue space cover are given compared to the reference category (<1 % blue space cover), associations of ≥5 % blue space cover are given compared to the reference category (<5 % blue space cover). Models included percent park cover, NDVI, percent blue space cover and were adjusted for calendar year, region, US census covariates, % ever smoked, , an offset for total person-time and strata for all possible combinations of sex, race, Medicaid Eligibility, age at study entry (2-year categories), and follow-up year. Urban cohorts included all person years in zip codes with a population density of 1000+ persons/mile^2.

© 2022 Klompmaker JO et al. *JAMA Network Open*

eTable 5. Total Number of Alzheimer Disease and Related Dementias and Parkinson Disease Hospital Admissions Avoided in 1 Year[a]

Exposure	ADRD hospitalization		PD hospitalization	
	Full cohort	Urban cohort	Full cohort	Urban cohort
	Number Avoided (95% CI)	Number Avoided (95% CI)	Number Avoided (95% CI)	Number Avoided (95% CI)
**NDVI**	11794 (9400, 14201)	663 (-1112, 2452)	2087 (1680, 2497)	898 (590, 1210)
**% Park cover**	2103 (1100, 3108)	970 (63, 1880)	900 (714, 1087)	239 (74, 405)
**≥1 % Blue space cover**	1527 (34, 3031)	-252 (-1344, 852)	995 (701, 1291)	278 (67, 493)

[a.] The estimated total number of hospital admissions avoided among this population in one year if, hypothetically, zip code NDVI (0.14) and park cover (7.9) increased by 0.5 IQR, and blue space cover increased to ≥1 % in all zip codes in the US. We used HRs of the main model to calculate the total number of ADRD and PD hospital admissions avoided in one year (Table 2).

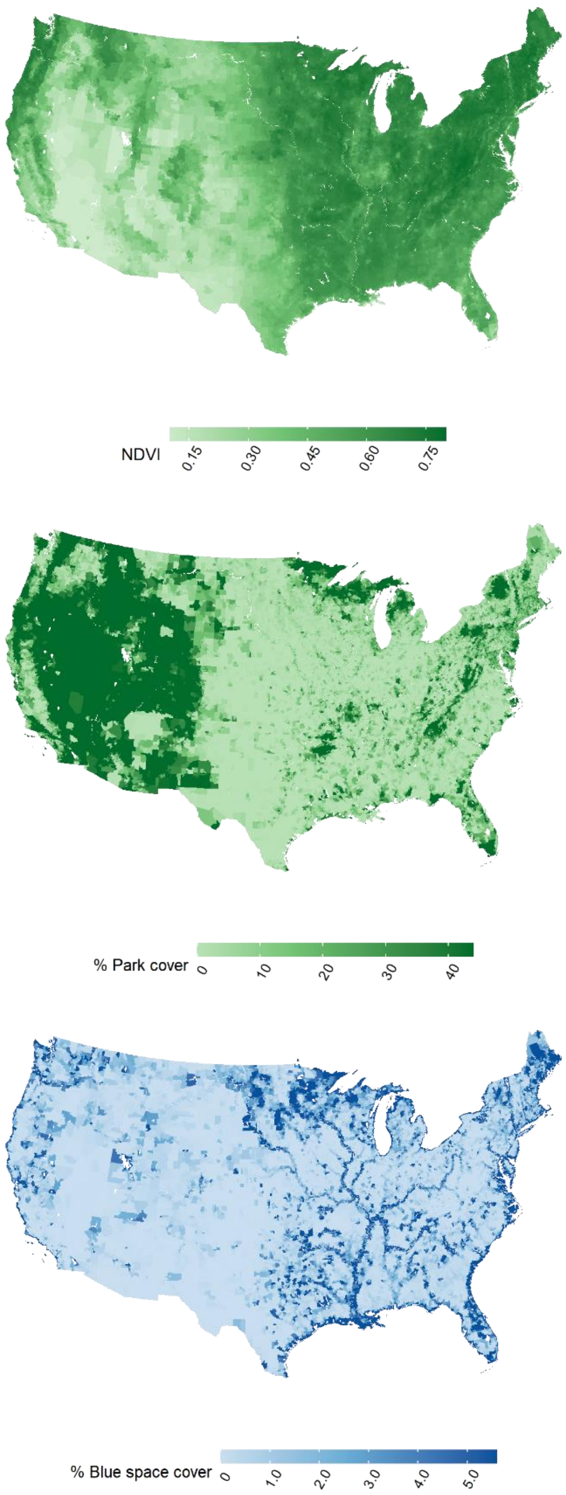

eFigure 1. Spatial Variation of Zip Code−Level Normalized Difference Vegetation Index (2008), Percent Park Cover, and Percent Blue Space Cover in the Contiguous US[a]

[a] To aid in visualization, the % Park cover map was truncated at 44.0%, the NDVI map was truncated at 0.1 and 0.8 and the % Blue space cover map was truncated at 5.6%

© 2022 Klompmaker JO et al. *JAMA Network Open*

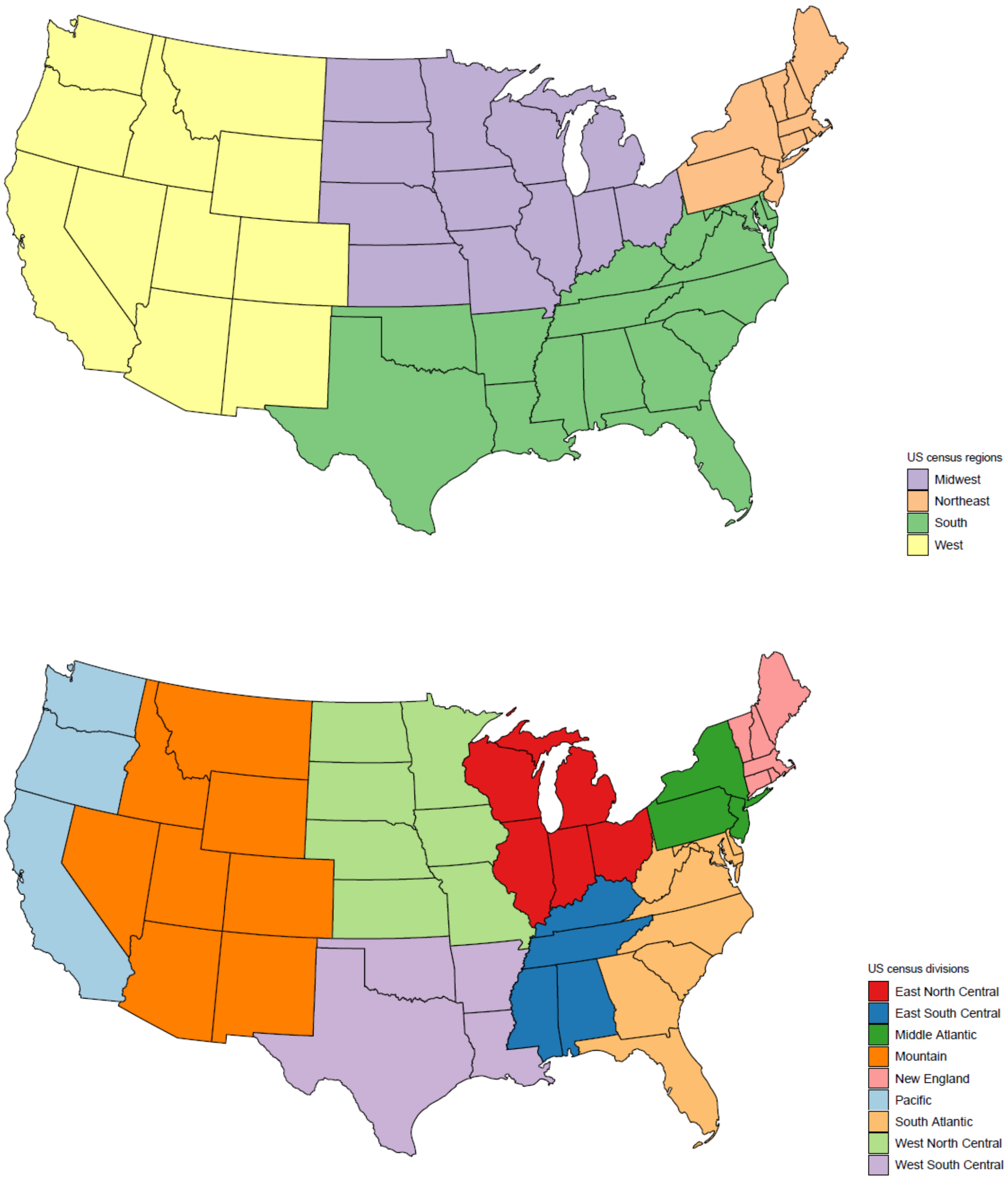

eFigure 2. US Census Regions and US Census Divisions

© 2022 Klompmaker JO et al. *JAMA Network Open*

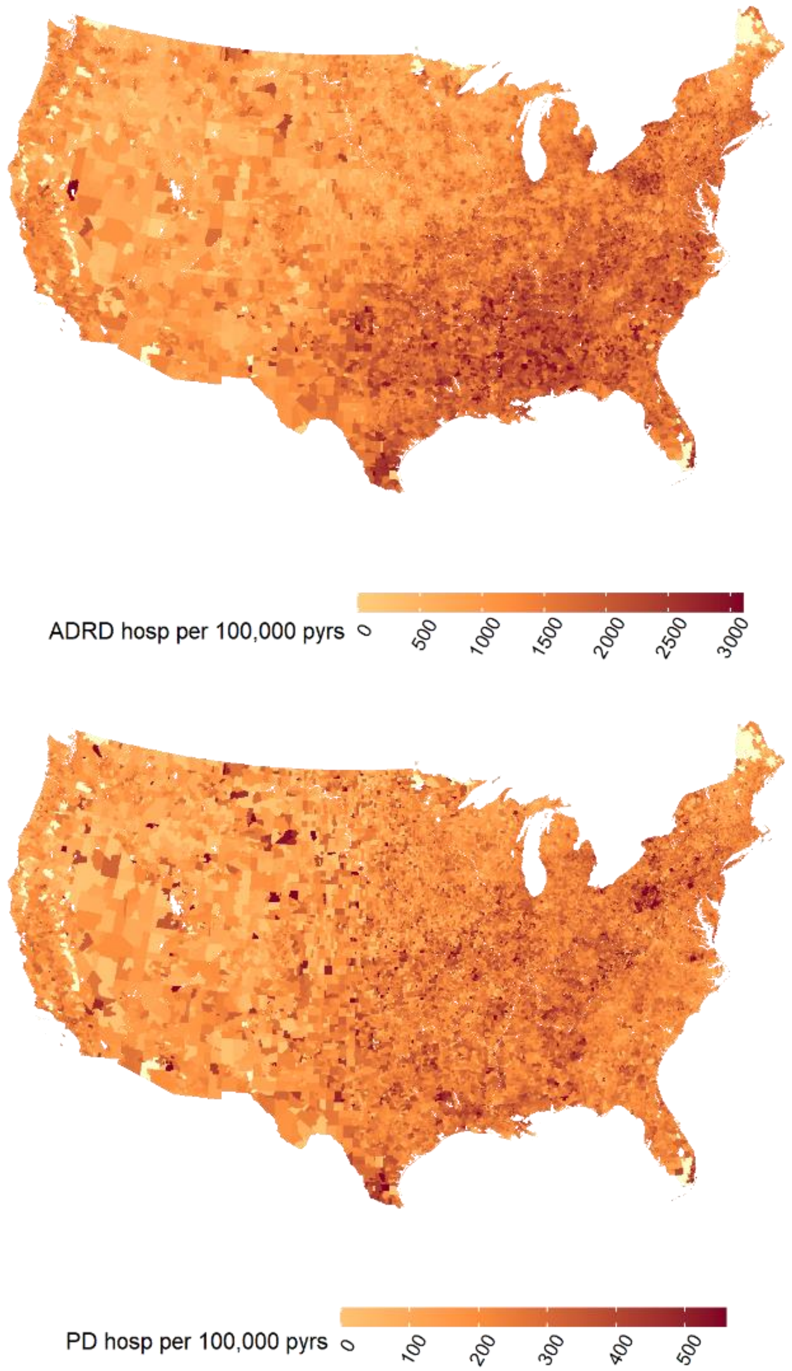

eFigure 3. Maps of Alzheimer Disease and Related Dementias and Parkinson Disease Hospitalizations per 100,000 Person-Years, 2000 to 2016[a]

[a] hosp = hospitalization, pyrs = person years

© 2022 Klompmaker JO et al. *JAMA Network Open*

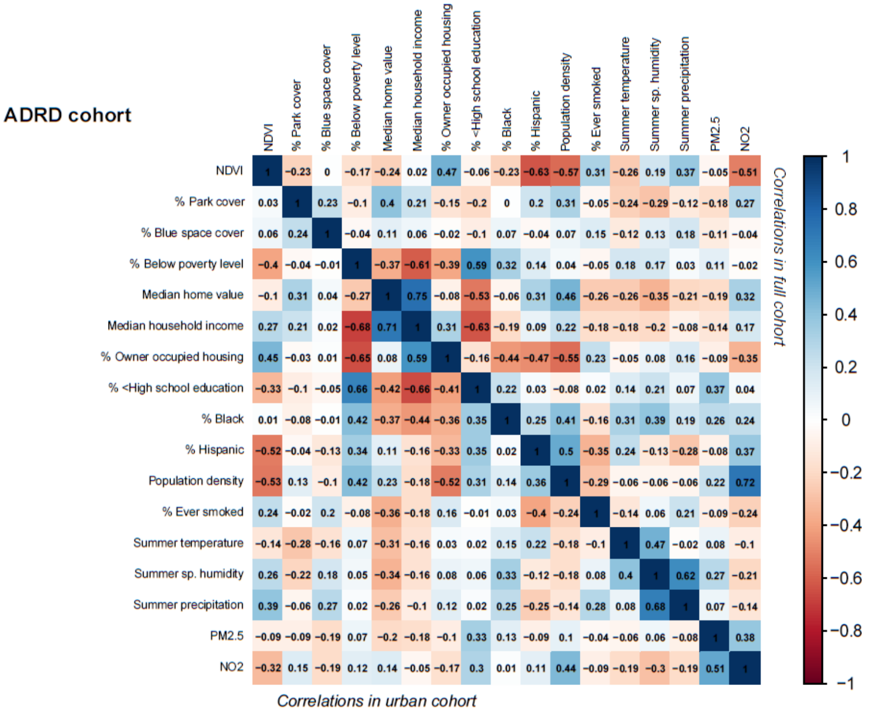

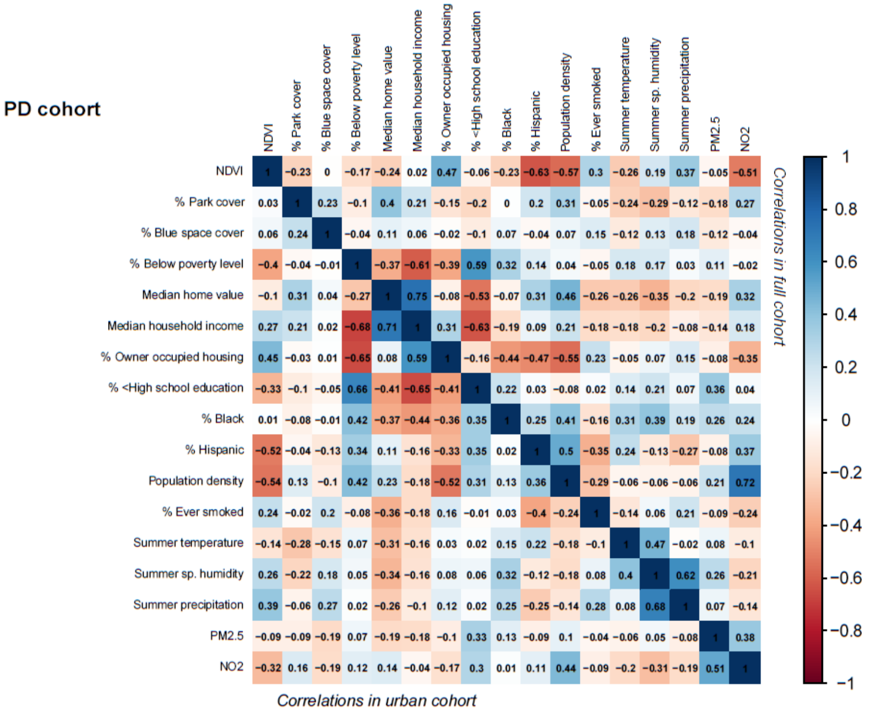

eFigure 4. Spearman Correlations Among Normalized Difference Vegetation Index, Percent Park Cover, Percent Blue Space Cover, and Other Covariates[a]

[a] Correlations above the diagonal are for the full population, correlations below the diagonal are for the urban population. Urban cohorts included all person years in zip codes with a population density of 1000+ persons/mile2.

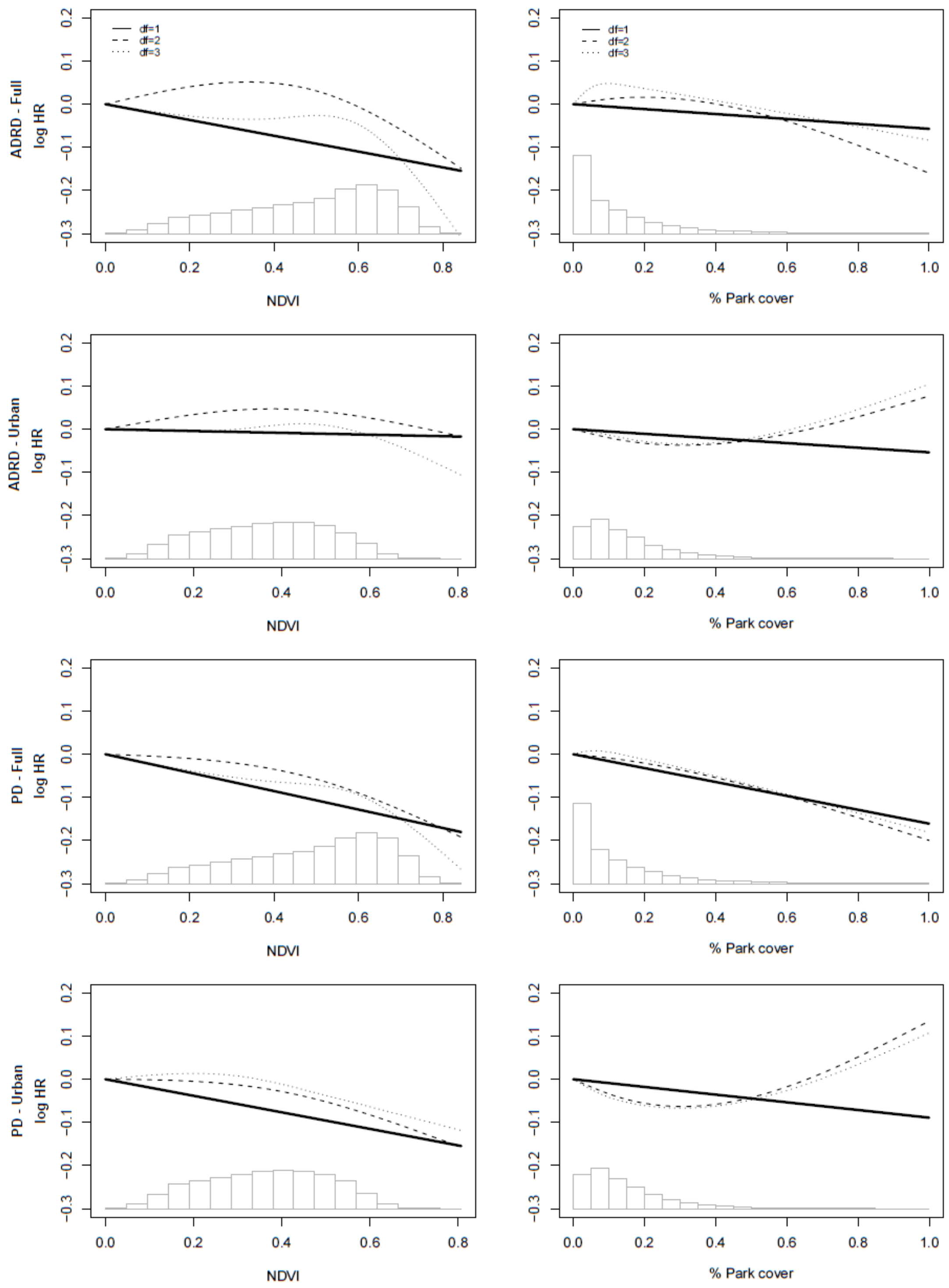

eFigure 5. Exposure-Response Curves (1, 2, or 3 Degrees of Freedom) of Normalized Difference Vegetation Index and Percent Park Cover With Alzheimer Disease and Related Dementias and Parkinson Disease Hospitalization[a]

[a] Models included NDVI, percent park cover, percent blue space cover and were adjusted for calendar year, region, US census covariates, % ever smoked, an offset for total person-time and strata for all possible combinations of sex, race, Medicaid Eligibility, age at study entry (2-year categories), and follow-up year. Urban cohorts included all person years in zip codes with a population density of 1000+ persons/mile^2.

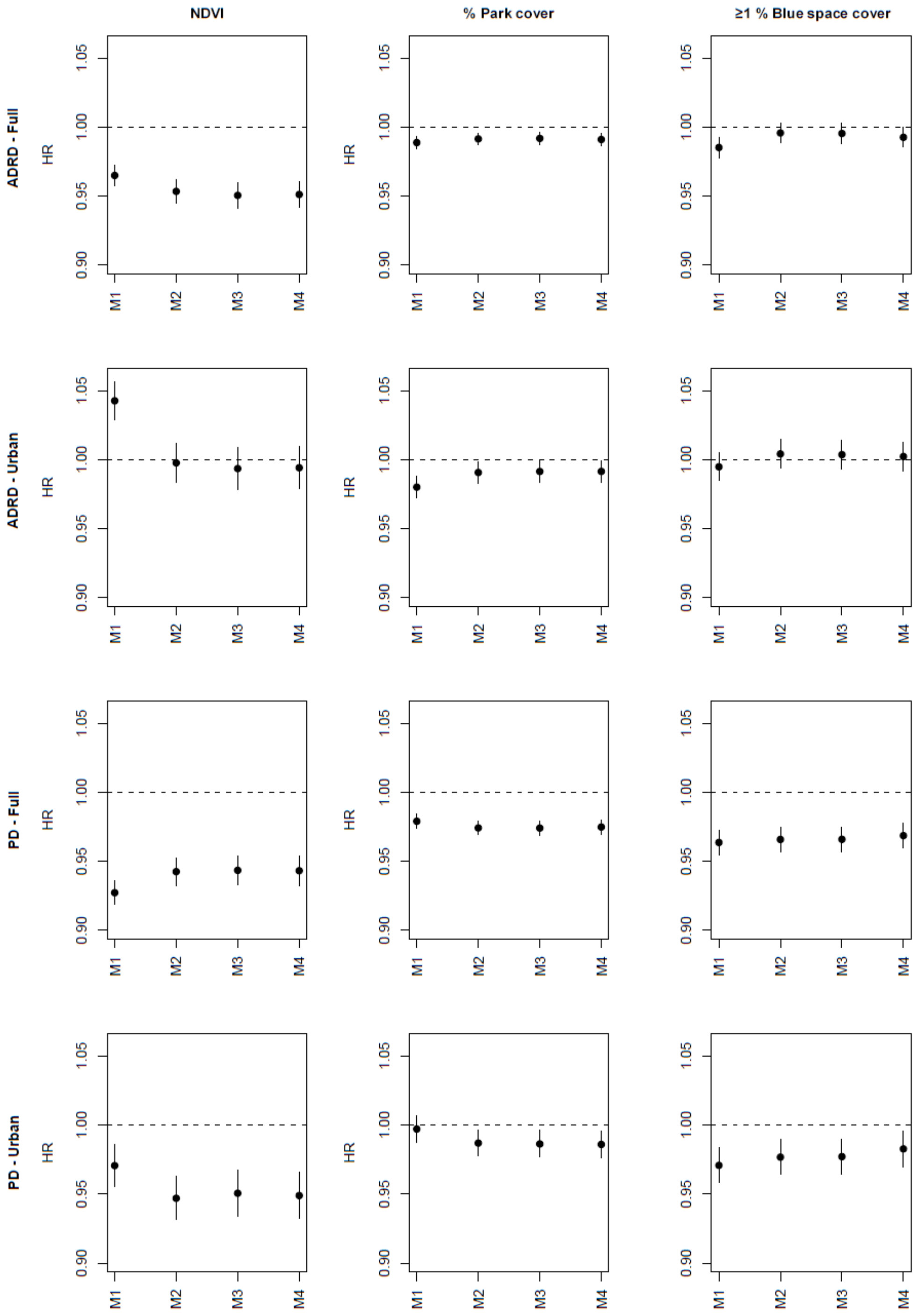

eFigure 6. Associations of Normalized Difference Vegetation Index, Percent Park Cover, and Blue Space Cover (≥1.0%) With Alzheimer Disease and Related Dementias and Parkinson Disease Hospitalization in Models With Increasing Adjustment for Potential Confounders[a]

[a] Associations of NDVI (0.27) and percent park cover (15.9) are expressed per IQR increase of the full cohort, associations of ≥1 % blue space cover are given compared to the reference category (<1 % blue space cover). Model 1 included NDVI, percent park cover, percent blue space cover, calendar year, region and strata for all possible combinations of sex, race, dual, age at study entry (2-year categories) and follow-up year. In Model 2 all US census covariates (except population density) were added. In Model 3 population density was added. In Model 4 % ever smoked was added. Urban cohorts included all person years in zip codes with a population density of 1000+ persons/mile^2.

© 2022 Klompmaker JO et al. *JAMA Network Open*

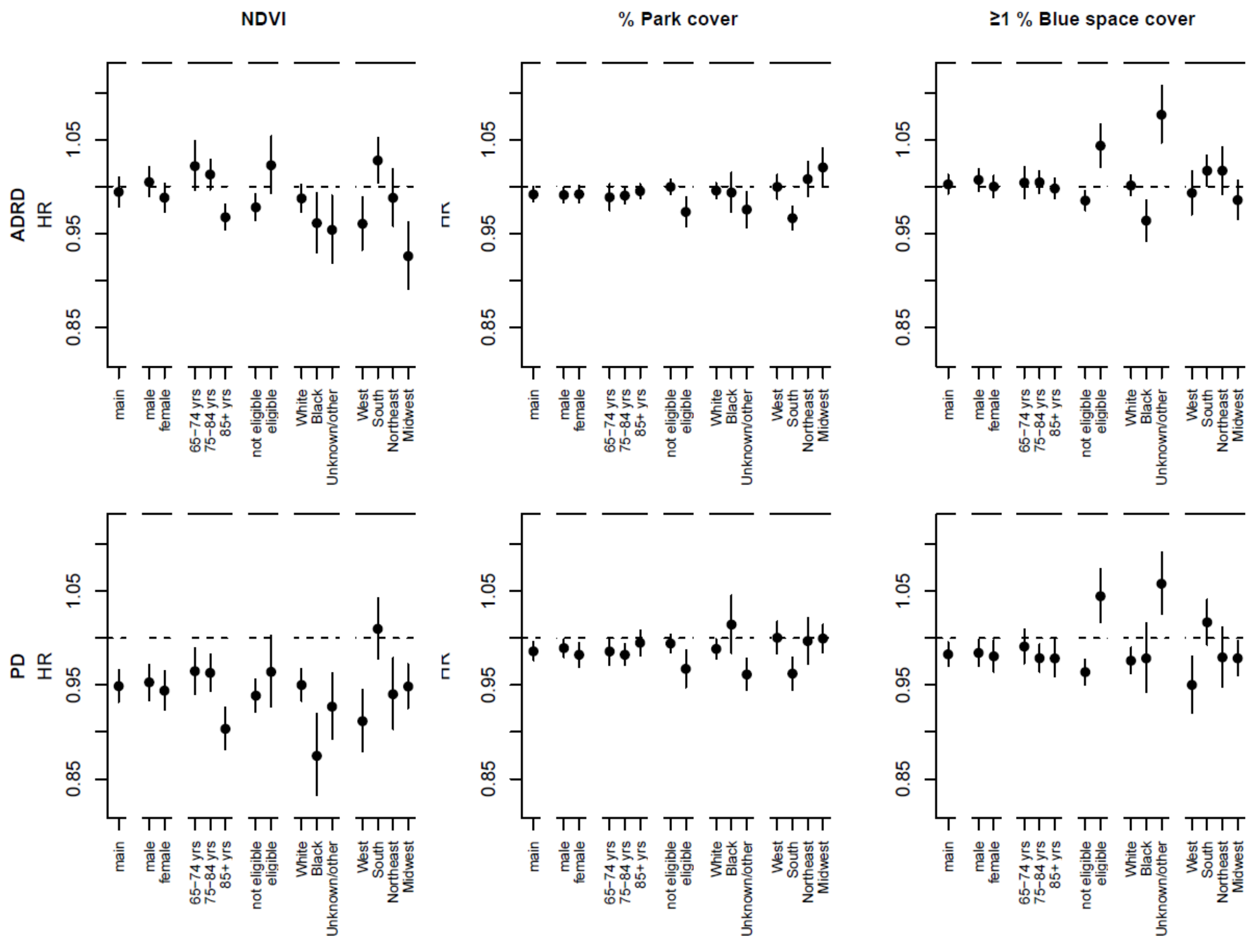

eFigure 7. Associations of Normalized Difference Vegetation Index, Percent Park Cover, and Blue Space Cover (≥1.0%) With Alzheimer Disease and Related Dementias and Parkinson Disease Hospitalization in the Urban Population in Stratified Analyses[a]

[a] Associations of NDVI (0.27) and percent park cover (15.9) are expressed per IQR increase of the full cohort, associations of ≥1 % blue space cover are given compared to the reference category (<1 % blue space cover). Models included NDVI, percent park cover, percent blue space cover and were adjusted for calendar year, region, US census covariates, % ever smoked, an offset for total person-time and strata for all possible combinations of sex, race, Medicaid Eligibility, age at study entry (2-year categories), and follow-up year. Urban cohorts included all person years in zip codes with a population density of 1000+ persons/mile^2.

© 2022 Klompmaker JO et al. *JAMA Network Open*

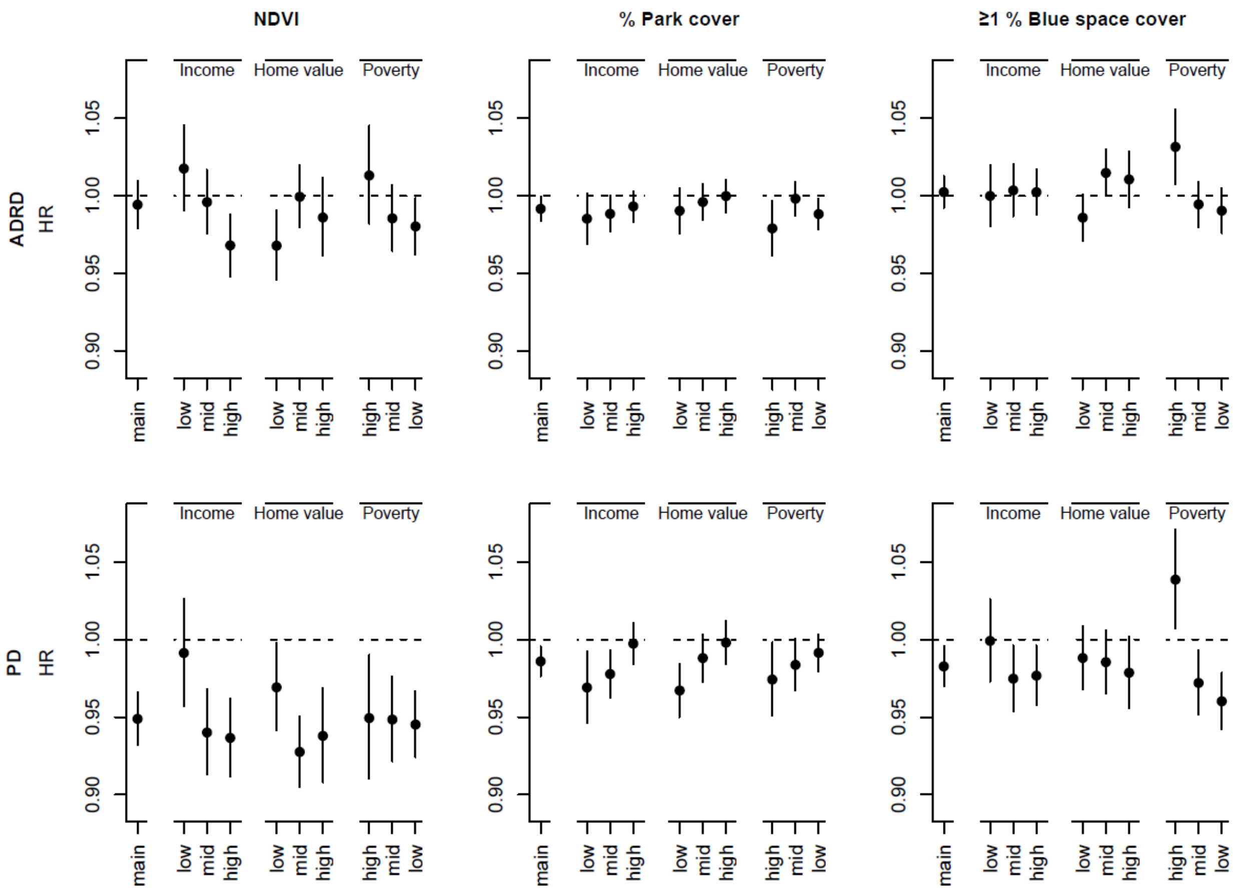

eFigure 8. Associations of Normalized Difference Vegetation Index, Percent Park Cover, and Blue Space Cover (≥1.0%) With Alzheimer Disease and Related Dementias and Parkinson Disease Hospitalization in the Urban Population in Stratified Analyses by Neighborhood Socioeconomic Status[a,b]

[a] Associations of NDVI (0.27) and percent park cover (15.9) are expressed per IQR increase of the full cohort, associations of ≥1 % blue space cover are given compared to the reference category (<1 % blue space cover). Models included NDVI, percent park cover, percent blue space cover and were adjusted for calendar year, region, US census covariates, % ever smoked, an offset for total person-time and strata for all possible combinations of sex, race, Medicaid Eligibility, age at study entry (2-year categories), and follow-up year. Urban cohorts included all person years in zip codes with a population density of 1000+ persons/mile[2].

[b] To define strata, we used the following quantiles (q33.3, q66.7) for the ADRD cohort: median household income ($1,000): 42.6, 62.5; median home value ($1,000): 146.8, 276.5; percent below the poverty level (%): 6.5, 11.6; for the PD cohort: median household income ($1,000): 42.6, 62.5; median home value ($1,000): 146.9, 276.4; percent below the poverty level (%): 6.5, 11.6.

© 2022 Klompmaker JO et al. *JAMA Network Open*
