# Supplementary material for: Associations of Greenness, Parks, and Blue Space With Neurodegenerative Disease Hospitalizations Among Older US Adults
Source: JAMA Netw Open. 2022 Dec 20;5(12):e2247664. doi: 10.1001/jamanetworkopen.2022.47664 (PMC9856892; doi:10.1001/jamanetworkopen.2022.47664)
Supplement: Supplement 2. — Data Sharing Statement [file jamanetwopen-e2247664-s002.pdf]

Data Sharing Statement

Klompmaker. Associations of Greenness, Parks, and Blue Space With Neurodegenerative Disease Hospitalizations Among Older US Adults. *JAMA Netw Open*. Published December 20, 2022. doi:10.1001/jamanetworkopen.2022.47664

Data
Data available: No
